# A Longitudinal Study of *Mycobacterium avium* Subspecies *paratuberculosis* Infections in Cattle Using Lipid Antigens and Providing Evidence for Age-Associated Antibody Dynamics

**DOI:** 10.3390/ani16162611

**Published:** 2026-08-20

**Authors:** Valerie Hughes, Paul S. Mason, Anna Cervi, Dafydd A. Thomas, George Caldow, Karen Stevenson, Mark S. Baird

**Affiliations:** 1 Moredun Research Institute, Pentlands Science Park, Bush Loan, Penicuik EH26 0PZ, UK; val.hughes@moredun.ac.uk (V.H.);; 2 Diagnostig Ltd., MSParc, Gaerwen, Ynys Mon LL60 6AG, UKa.cervi@diagnostig.com (A.C.); d.a.thomas@diagnostig.com (D.A.T.); 3 Scotland’s Rural College, Peter Wilson Building, The King’s Building, West Mains Rd., Edinburgh EH9 3JG, UK

**Keywords:** Johne’s disease (JD), *Mycobacterium avium* subspecies *paratuberculosis* (MAP), serological tests, mycolic acids (MA), TDM, TMM, longitudinal, ELISA

## Abstract

**The problem:** Johne’s disease, caused by infection with *Mycobacterium avium* subspecies *paratuberculosis* (MAP), can be widespread in cattle herds. As a consequence of the slow progression of the infection and the delayed appearance of a humoral response, at any time few of the infected animals may be detected as positive with current serological tests. Many disease control initiatives have focused on removing infected animals by using serology to detect antibodies, principally to a purified MAP extract. However, the delayed humoral response to MAP extract, which can be preceded by shedding MAP in the faeces, limits the value of this control method. **The approach:** We used a longitudinal study to evaluate the timing and magnitude of antibody responses to lipid antigens. **The method:** Thirty breeding females from a beef cow herd with a history of Johne’s disease were recruited at 10–12 months old. Blood (serum) and faecal samples were collected at six-month intervals over five years. The serum samples were evaluated by enzyme-linked immunosorbent assay (ELISA) using six synthetic lipid antigens, each from a different class of chemical compound that are components of the mycobacterial cell wall. The responses were correlated with MAP infection status as determined by faecal culture, a commercial serum antibody ELISA (ID Screen^®^), and interferon-γ release assays. Nineteen animals were positive using either culture or ID Screen^®^ at one or more time points; eleven animals were never positive in either assay. **Results:** In the lipid ELISA, the median responses for all animals were significantly higher at time point 5 (when the animals were three years old) than those at earlier time points. The timing of this response is compatible with, but does not prove, a shift in immune-response profile. For the antigens MOD171 and RT237F2, the responses for each individual animal were higher at time point 5 than at time point 4. **Outcome**: The increase in response to lipid antigens MOD171 and RT237F2 between the ages of two and four years may indicate infection with MAP at an earlier stage than is achieved with the established serology tests.

## Simple Summary

Johne’s disease (JD), caused by *Mycobacterium avium* subspecies *paratuberculosis* (MAP), is widespread in cattle and other animals. The organism can contaminate the food chain, and because of its association with Crohn’s disease, there are widespread efforts to reduce the level of MAP infection in herds. JD is a chronic progressive wasting disease that is difficult to detect in the early stages of infection using serological methods generally targeted at antibodies to mycobacterial proteins. These are often supported by the detection of the organism in faeces by culture or PCR. While the current serology assays have high specificity, they miss many animals deemed to be positive by other detection methods and they are particularly poor at detecting infection in young animals. In this work, we evaluated a cohort of cows from a confirmed MAP-infected herd that were tested every six months over five years. We used serology assays with six lipid antigens, derivatives of synthetic mycolic acids (MA) identical to mycobacterial cell components. Every animal showed an increased response to two lipid antigens between 30 and 36 months of age. This novel test may determine whether an animal has been infected with MAP early in life. There was a correlation between high responses and faecal culture positivity (as recorded at any point in the study).

## 1. Introduction

*Mycobacterium avium* subspecies *paratuberculosis* (MAP) causes Johne’s disease (JD) in ruminants, camelids and leporids and can infect a wide range of animals [1,2,3,4,5,6]. Pathogenesis manifests as an infection of intestinal macrophages, with MAP evading/suppressing the immune response and leading to a progressive protein-losing enteropathy, emaciation and finally death. Because of the slowly progressive nature of the disease, clinical signs first appear in middle-aged animals (usually older than two years), though the disease can occasionally occur in younger animals [7].

JD is associated with lower economic yields and negatively impacts animal welfare [8,9]. Moreover, the organisms are present in the milk of infected animals and may be incompletely inactivated by pasteurisation, which could pose a potential route of exposure to MAP for the human population [10,11]. Within the context of this report, exposure is taken to mean contact with MAP; infection refers to when MAP crosses the intestinal wall and interacts with the host, which triggers a detectable immune response.

MAP is more frequently found in the tissues of patients with Crohn’s disease than in those without the disease [12], but causality has not been proven. Nevertheless, the potential human health impact is one of the drivers for herd-level control of this infection, particularly in dairy cattle, and underlines the importance of reliable early diagnosis of JD [13].

While there is little evidence of absolute age resistance, until 12 months of age, younger calves are infected, and the higher the infectious dose, the more likely there is to be progression towards clinical disease [14]. Once infection has occurred, the disease develops slowly in cattle, and four stages have been described [7,15]. However, there is not a defined progression through these stages; even if an animal is ‘infected’, i.e., organisms persist in tissues after ingestion, it may be possible for it to overcome the infection by activation of its immune response, which could lead to the killing of all invading cells, or alternatively for MAP cells to become latent within a granuloma [16]. Moreover, it has been proposed that for every late-stage animal on a dairy farm, 15 to 25 other animals are infected [17], though this figure may be too high [18]. Thus, while infection is widespread in affected herds, as indicated under experimental conditions, not all animals develop persistent infection, nor do all infected animals shed MAP in their faeces or progress to clinical disease [19]. The increased susceptibility of young animals to infection is important to consider when designing strategies to reduce transmission. The potential for faecal-oral transmission on an infected commercial farm is high. Additionally, both milk and colostrum can be a source of infection. Together, these ensure that in any infected herd, the risk of infection for young animals is high [7,20,21,22].

Diagnosis of JD is confounded by the complexities of MAP’s pathogenesis. Histopathology provides a reliable way to detect developing disease, but it can only be determined post-mortem and requires expert interpretation, which can be subjective [23]. Tissue culture can also reliably detect MAP infection in animals that are intermittent faecal shedders [15,24].

The direct passage of MAP through the gut following ingestion (passive shedding) may result in positive faecal screens that are not associated with an infection [15]. It is difficult to estimate the proportion of positive results attributable to passive shedding, and few studies have attempted to address this. Moreover, intermittent shedding patterns also contribute to the lack of reproducibility of this diagnostic method [25] and there is limited direct evidence to identify the balance of shedding and non-shedding in infected animals, the pattern of which is likely to be dependent on the animal and its environmental pressures [7,26]. In addition, the use of PCR detection methods must also be treated with caution, as dead organisms may be detected. In a recent study, the recommended maximum number of PCR cycle counts that should be interpreted as indicative of ‘infection’ has been reduced from <37 to <33, with any >40 being counted as ‘infection excluded’ [27]. Faecal tests are also relatively expensive for annual whole-herd screening. Moreover, it is not known whether young animals that test positive by faecal culture are more likely to develop disease or to shed MAP at levels sufficient to cause new infections.

The current widely used commercial serological tests for antemortem diagnosis generally provide excellent specificity, but very low sensitivity [27]. These assays are most effective in the later stages of the disease, when animals may have been infectious for some time [28]. It has been suggested that the early stages of MAP infection are dominated by protective Th1 responses, and that this then switches to Th2 dominance, the so-called classical switch profile [29,30,31,32,33,34,35,36,37], possibly triggered by stress during lactation or parturition. Whatever the trigger, the progression from subclinical to clinical disease may coincide with this shift. MAP antigen-stimulated interferon gamma (IFN-γ) production has been shown to be elevated in the Th1 stage of subclinical infection [38], but this cytokine declines in the clinical stage concomitant with an increase in MAP-specific IL-10 production [39]. Elevated responses to IFN-γ can provide an effective measure of herd infection with MAP [40]. However, in longitudinal studies of artificially infected animals [41,42] or naturally infected animals [40], both Th1 (IFN-γ) and Th2 (IgG1 antibody) responses can occur concomitantly in early-stage disease.

There is often a poor correlation between current commercial serological tests and the detection of MAP in faeces [43,44,45,46,47]. The use of combinations of these assays provides a more reliable diagnosis [48], but the ability to identify cattle in the early stages of infection, before they are infectious, and to support clear herd management decisions remains a major challenge [49,50,51]. Thus, as current control programmes drive down the numbers of later-stage infective animals in herds, further progress in reducing disease prevalence requires reliable methods for early detection of infection [52].

**Figure 1:**
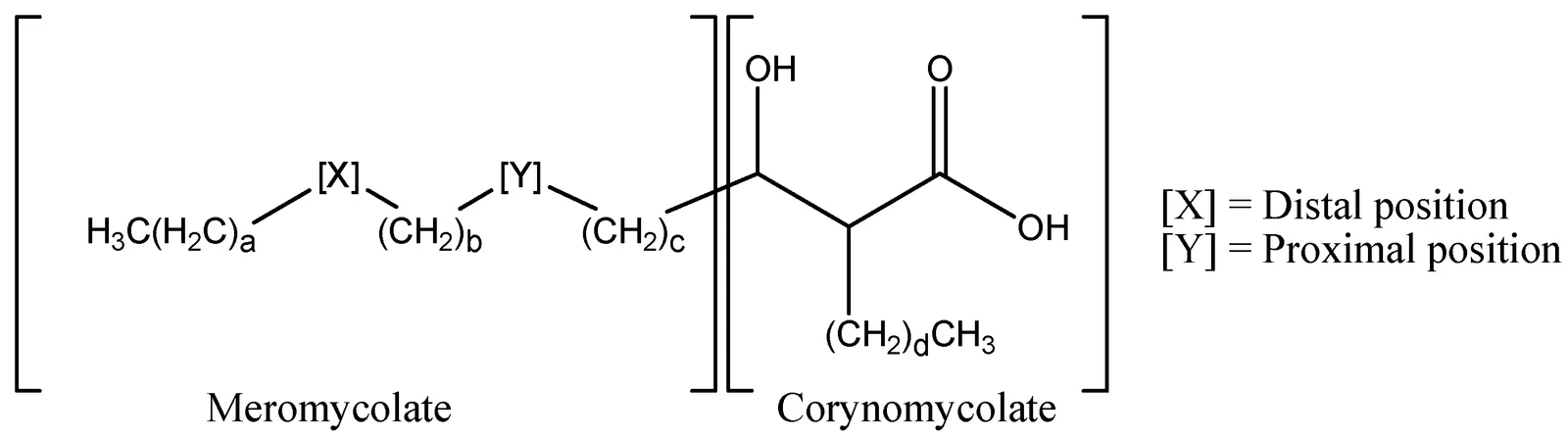
The generic structure of mycolic acids (MA).

We have previously reported an alternative serological approach using synthetic lipid antigens. These are based on characteristic long-chain β-hydroxy-fatty acids, mycolic acids (MA, Figure 1), identical to individual components of complex mixtures that are present in the cells of mycobacteria, including MAP. The natural mixtures comprise molecules with a variety of X and Y groups and chain lengths a–d.

They are often present in cells as sugar esters (ME), in particular trehalose mono- and di-mycolates (TMM and TDM) [53], or bound to the cell wall sugars.

An exploratory study showed that an enzyme-linked immunosorbent assay (ELISA) using such MA-based antigens detected MAP infection, as defined by the established tests of IDEXX serum ELISA for MAP and by faecal PCR. The assay evaluated six antigens from different chemical subclasses of MA or ME to determine the best individual responses and then used a combination of responses to optimise sensitivity and specificity. The lipid ELISA also identified a number of PCR-positive animals that were not detected by the IDEXX serum ELISA [53], and the results were not affected by vaccination either against bovine tuberculosis (BTb) or JD, or by BTb infection.

The present study was carried out to determine whether this MA-based assay can provide useful information on the dynamics of the immune response to MAP, and whether it can identify infected animals ahead of current commercial MAP serological tests or faecal detection. We have applied the lipid ELISA to a set of longitudinal samples from animals in a MAP-infected herd, which had been monitored by a conventional JD ELISA and by a MAP antigen-driven IGRA [40]. This provides the means to investigate the timing of the response to specific antigens, and whether this response to different classes of lipid antigen may change as the disease progresses, in the same way that, in tuberculosis, the balance of specific MA classes (and the stereochemistry within the classes) is known to change with disease progression [54].

## 2. Materials and Methods

### 2.1. The Longitudinal Study Farm (High Risk for Paratuberculosis)

The beef cattle herd selected for the longitudinal study was from a farm in Scotland participating in the national paratuberculosis control programme since 1999. In accordance with this scheme, all breeding stock over two years of age were tested annually for paratuberculosis by serum ELISA. Throughout this study, the status of the herd was Johne’s risk level 3 (meaning that annually up to 3% of the herd tested positive by serum ELISA) [40].

Replacement breeding heifers were recruited at 10–12 months of age from the study farm (a mix of Aberdeen Angus cross and Salers cross). Animals remained on the farm until the end of the study unless removed from the herd for management or disease control. The farm manager made decisions about when to remove animals from the herd, but the farm policy was to remove any animal once it tested positive on ID Screen^®^ or failed to conceive [40].

Bovine tuberculosis testing was as described earlier [40] but is described in full in Supplementary S1.

Sample collection on the longitudinal study farm has been described earlier [40] and is detailed in Supplementary S2. Briefly, faeces and blood samples were collected at six-month intervals; serum samples were prepared from the blood and stored at −40 °C. The samples from each collection are identified as TP1 for the first collection, to TP11. A set of samples labelled TP10a was taken a week after a single intradermal comparative cervical tuberculin test (SICCT) for BTb was carried out, while those labelled TP10 were taken before the skin test. Samples with odd TP numbers were collected in the summer, and those with even numbers in the winter.

### 2.2. Antigens

Six antigens were evaluated in this work, each from a different class of MA or ME. Their structures are presented in Supplementary Figure S1. The antigen MOD171 is based on a cell-wall-bound MA fragment, a triarabinose dimycolate, incorporating a major α-MA present in many mycobacteria, including organisms of the *Mycobacterium avium* complex (MAC) [55]. ZAM295 is a trehalose dimycolate of a diene-MA, a sub-group commonly present in non-tuberculous mycobacteria [56]. ST123 is a trehalose dimycolate of a wax ester; this class of molecule is present in MAC, replacing the keto-MA class present in many other mycobacteria [54,57,58]. SMP70 is a glucose monomycolate of a common methoxy-MA [59]. RT237F2 is an ester of a major α-MA with an amino-sugar; it is not a known component of mycobacteria, but a simpler analogue of a mycolyl muramyl dipeptide [60]. JRRR121 is a free methoxy-MA, incorporating a *trans*-cyclopropane fragment; such *trans*-cyclopropanes are major components of the methoxy-MA class present in MAC [61].

### 2.3. Culture, Serum ELISA (ID Screen^®^) and IGRA Analysis

Detection of MAP in faecal samples was carried out as previously reported [40]. Faecal samples were decontaminated by a slightly modified double incubation method as described [62]. Briefly, 2 g of faeces were mixed with 35 mL sterile deionised water. Particulate material was allowed to settle for 30 min, and the top 5 mL of the liquid fraction was removed and treated with hexadecylpyridinium in half-strength Brain Heart Infusion Broth (Oxoid Ltd., Hampshire, UK) to a final concentration of 0.7% (*w*/*v*) at 37 °C for 18 h. The suspension was centrifuged at 1500× *g* for 30 min. The resulting pellet was resuspended in 1 mL para-JEM AS (ThermoFisher Scientific, Paisley, UK, reconstituted in accordance with the manufacturer’s protocol and diluted 15-fold in Brain Heart Infusion Broth) and incubated for a further 18 h at 37 °C. This was then used to inoculate broth (reconstituted from para-JEM Reagent Kit w/Blue, in accordance with the manufacture’s protocol) and cultured using the TREK ESP II system for detection of MAP [63]. Samples signalling positive were submitted for Ziehl–Neelsen staining to confirm the presence of acid-fast bacteria, and the presence of MAP was confirmed by PCR [64].

For some analyses, the samples were divided into three groups: those from animals never positive by either culture or ID Screen^®^ (G0), those positive by either at any time point other than TP1 (G1), or those positive by culture alone and only at TP1.

### 2.4. Lipid ELISA

Antibody concentrations were measured in serum samples that had been frozen until the point of assay. All samples were evaluated in ELISA, using six separate synthetic lipid antigens, comprising different classes of MA and ME, as described in Section 2.2.

The ELISA was carried out using the same method as described earlier [53] on 96-well microplates, and the purified antigens were dissolved in THF: n-hexane (1:50, 5 mL) at a concentration of 5.6 μM; 50 μL of this antigen solution was placed in each well. The plates were left to dry at room temperature overnight. To reduce non-specific hybridisations, the plates were blocked with casein/PBS containing 0.5% *w*/*v* casein (300 μL/well) and incubated at 25 °C for 30 min. The solution was aspirated using the LT-3500 plate washer, and the plates were flicked dry. The serum (1:40 dilution in casein/PBS; 50 μL/well) was added to the plate and incubated at 25 °C for 1 h. The serum was aspirated and the plates washed three times with casein/PBS (400 μL/well) using the same plate washer, and the plates were again flicked dry. The secondary antibody (Peroxidase-AffiniPure Goat Anti-Bovine IgG, Stratech Fc; 1:1000 dilution in casein/PBS) was added to the plate (50 μL/well) and left to incubate at 25° C for 30 min. The plates were again washed three times with casein/PBS (400 μL/well) and dried. OPD substrate (0.2 mg/mL) was added and the plates (50 μL/well) incubated at 25° C for 30 min; the reaction was then stopped by the addition of 2.5 M H_2_SO_4_ (50 μL/well), and the absorbances of each well were read straight away at 492, 450 and 620 nm using an LT-4000 plate reader; the values at 492 nm were used for the current analysis.

Typically, two replicate samples were analysed. The average optical densities (OD) (492 nm) of the replicates and their standard deviations were recorded. On each plate, a common pooled positive control (using samples from animals positive to both faecal PCR and IDEXX serum JD ELISA) and a PBS/casein negative control were run alongside test serum samples. Whenever a pooled positive control had to be updated, it was standardised against the existing control. It was also compared to a commercial positive standard obtained from GD Animal Health (Royal GD, P.O. Box 9, 7400 AA Deventer, The Netherlands). The positive control was used to normalise OD readings on different plates by converting readings for individual wells into a percentage of the pooled positive response.

### 2.5. Statistical Analysis

Statistical analysis was carried out using R version 4.2.2 (2022-10-31 ucrt) [65]. The significance of differences between the data for each cohort of samples was initially determined using ANOVA, followed by the Tukey’s test. However, in most cases, Q-Q plots were not linear. Further analysis was therefore carried out using ANOVA on log-transformed (ln) data, when Q-Q plots for most lipid antigens were linear. However, to provide consistency across all assays, the data were then analysed using the Kruskal–Wallis approach, followed by false discovery rate (FDR) analysis to correct for significance. The responses for each sample with the six lipid antigens, faecal culture, the two IGRAs, and ID Screen^®^ are presented in the Supplementary Data, with a statistical comparison of the data for each time point with those for every other time point.

One animal was removed after the second time point and is not included in the statistical analysis. Moreover, two animals were removed before TP8 (one with a positive faecal culture, one with a positive ID Screen^®^), three before TP9 (one with 4 positive faecal cultures and a positive ID Screen^®^, one with 4 positive faecal cultures and two positive ID Screen^®^, one with one positive ID Screen^®^), and three before TP10 (one with 7 positive faecal cultures, one with 4 positive faecal cultures, one with one positive faecal cultures). Direct statistical comparison between data up to TP7 and those after this time must therefore be treated with considerable care. The animals removed therefore included many of those showing significant numbers of positive responses in faecal culture or ID Screen^®^.

Where cut-offs were used to define a positive sample for each antigen, optimal cut-offs to match samples positive to both IDEXX assay and PCR were derived from the earlier publication [53].

## 3. Results

The results of the lipid ELISA with each antigen, together with those for culture, ID Screen^®^, and two different IGRAs, are reported animal by animal in Supplementary Tables S1.1–S1.30.

Nineteen of the thirty animals were positive either by culture or by ID Screen^®^ at some time during the study:

- Sixteen showed a positive culture response at one or more times. One animal was culture positive at seven times (including TP1), two at four times, two at three times, two twice, and nine at one time. Of the nine single positives, five were at the first time-point.
- Six animals showed elevated scores for the serum ELISA (ID Screen^®^) above the recommended cut-off for a positive diagnosis (≥70%) (two others showed inconclusive responses between 65 and 70%). Of the six, three were also positive for faecal culture three or four times. The other three animals with a positive ID Screen^®^ response did not test positive by faecal culture at any time. This exemplifies the variable nature of the established tests and their failure to agree that an animal is MAP positive at any given time point.
- As specified in 2.5 above, a number of animals were removed beyond TP7 due to the management strategy on the farm. Many of these animals had positive PCR or ID Screen^®^ responses at some stage before they were removed. This clearly could affect the median responses for the remaining animals and the number of remaining animals above cut-offs in each assay. Thus, although the remaining animals at TP11 included nine with a positive PCR at any time, just two had any positive ID Screen^®^ results. Comparison of data for TP8 to TP11 with those earlier time points must therefore only be made with this in mind.

### 3.1. ELISA Responses with Antigen MOD171

**Figure 2:**
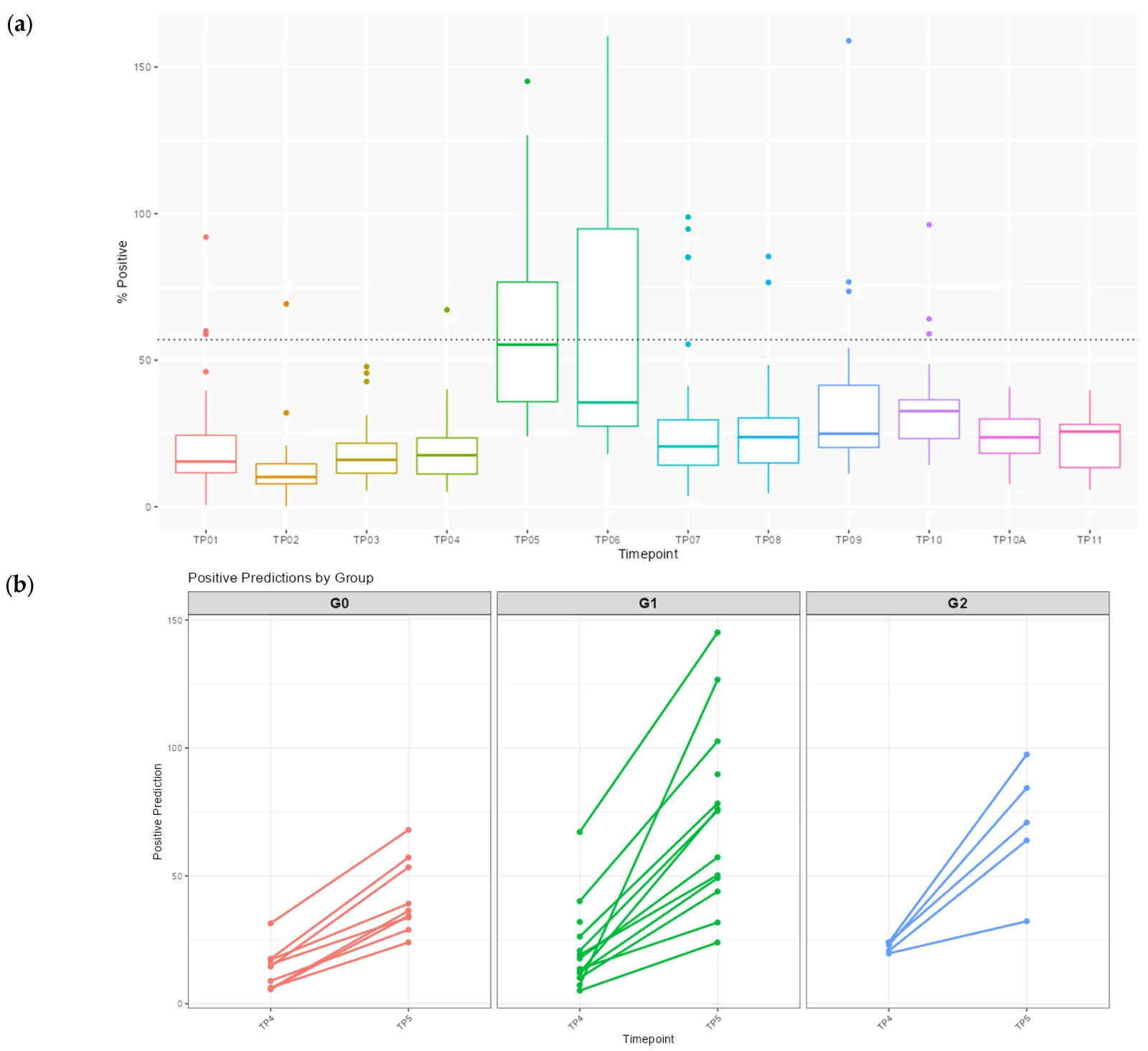
(**a**): Box-and-whisker plot of responses of thirty animals in the lipid assay with MOD171 at each time point (TP). The dotted line shows the cut-off for a positive assignment of MAP infection using antigen MOD171, based on earlier work [53]. (**b**): Change in ELISA response for each animal from TP4 to TP5 using MOD171 as antigen. G0 are animals that have never had a positive culture or ID Screen^®^ response. G1 are animals with a positive culture or ID Screen^®^ response at any time other than TP1; G2 are animals with a culture response at time point TP1 only, and no positive ID Screen^®^ response at any time. There is no statistically significant difference between the median increases in response (as a percentage response compared to a positive control) between the three groups. However, the response of group G0 at TP5 was significantly lower than that of G1 (Supplementary Table S2c) *p* < 0.005).

The ELISA results for the lipid antigen MOD171, a tri-arabinose dimycolate model for cell wall-bound α-MA, are shown as box-and-whisker plots in Figure 2a. Statistical analysis shows there was a highly significant increase in response from bleed TP4 (animals 28–30 months old) to TP5 (Table S2a,b) (*p* < 0.0001). Moreover, where it was possible to make a comparison, every single animal showed an increase from TP4 to TP5 (Figure 2b, Table S2a). There was then a trend for a reduction in response at TP6, although this was not significant, followed by a significant drop in response from TP6 to most subsequent time points (Table S2b). Nonetheless, some animals remain above the cut-off determined for a MAP positive test result [53] after TP6.

Seventeen of the animals gave responses with MOD171 at TP5 or TP6 above the cut-off determined for a positive response [53]. These included two of the three animals that were positive at any time by culture and also at any time by ID Screen^®^, and twelve of sixteen positive at any time by culture only.

As Figure 2b shows, with this antigen, all animals showed increased responses from TP4 to TP5. There was no significant difference in the increases between animals never positive by either culture or ID Screen^®^ (G0), those positive by either at any time point other than TP1 (G1), or those positive just by culture at TP1 (G2).

There was a significant drop in median responses between TP1 and TP2 (*p* < 0.005).

### 3.2. ELISA Responses with Antigen RT237F2

**Figure 3:**
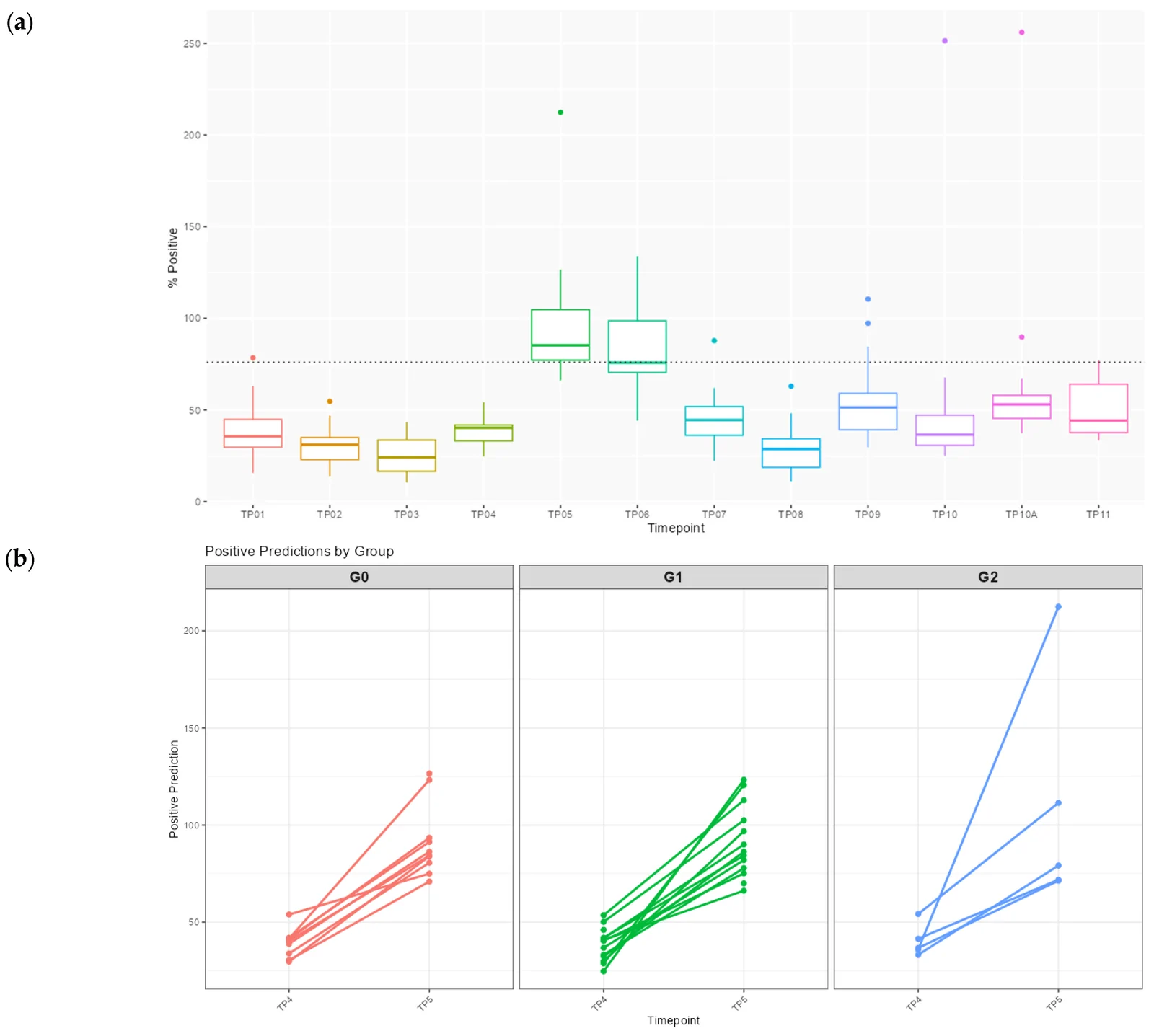
(**a**): Box-and-whisker plot of responses of thirty animals in the lipid assay with RT237F2 at each time point (TP). The dotted line shows the cut-off for a positive assignment of MAP infection using antigen RT237F2, based on earlier work [53]. (**b**): Change in ELISA response for each animal from TP4 to TP5 using RT237f2 as antigen. G0 comprises animals that never had a positive culture or ID Screen^®^ response. G1 comprises animals with a positive culture or ID Screen^®^ response at any time other than TP1; G2 comprises animals with a culture response at time point TP1 only, and no positive ID Screen^®^ response at any time. There is no statistically significant difference between the median increases in response (as a percentage response compared to a positive control) between the three groups, nor in the response of groups at TP4 or at TP5.

The antigen RT237F2 also showed similarly enhanced responses at TP5 (Figure 3a). In this case, the responses for both bleeds TP5 and TP6 were statistically higher than any of the other bleeds (*p* < 0.005). Moreover, none of the animals exceeded the cut-off determined for a positive sample at TP1–TP3. The average response for TP5 was 95% of the pooled positive control. There were also significant increases in response after the skin test at TP10. The increase in responses from TP4 to TP5 and TP6 is highly significant (Kruskal-Wallis), as is the drop after TP6. Nonetheless, some animals remain above the cut-off after TP6 (Table S3a,b).

As Figure 3b shows, with this antigen, all animals again showed increased responses from TP4 to TP5. There was no significant difference in the increases between animals never positive by either culture or ID Screen^®^ (G0), those positive by either at any time-point other than TP1, or those positive only by culture at TP1.

### 3.3. ELISA Responses with Other Lipid Antigens

**Figure 4:**
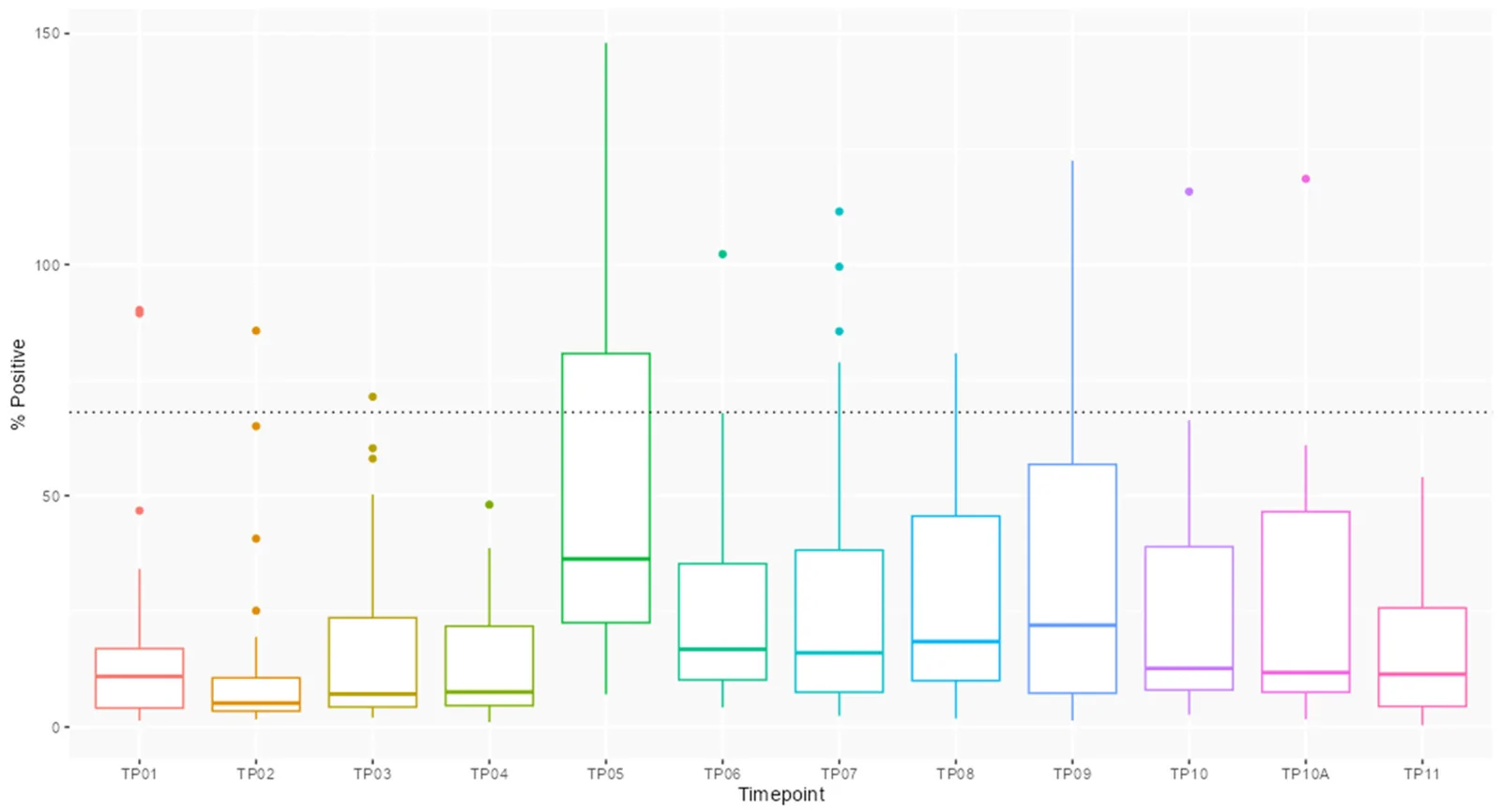
Box-and-whisker plot of responses of thirty animals in the lipid assay with ST123 as the antigen at each time point. The increase in median from TP4 (bleed 4) to TP5 and TP6 is highly significant, as is the drop from TP5 to TP6, though from TP6 on, there are no significant differences. The dotted line shows the cut-off for a positive assignment of MAP infection using antigen ST123, based on earlier work [53].

**Figure 5:**
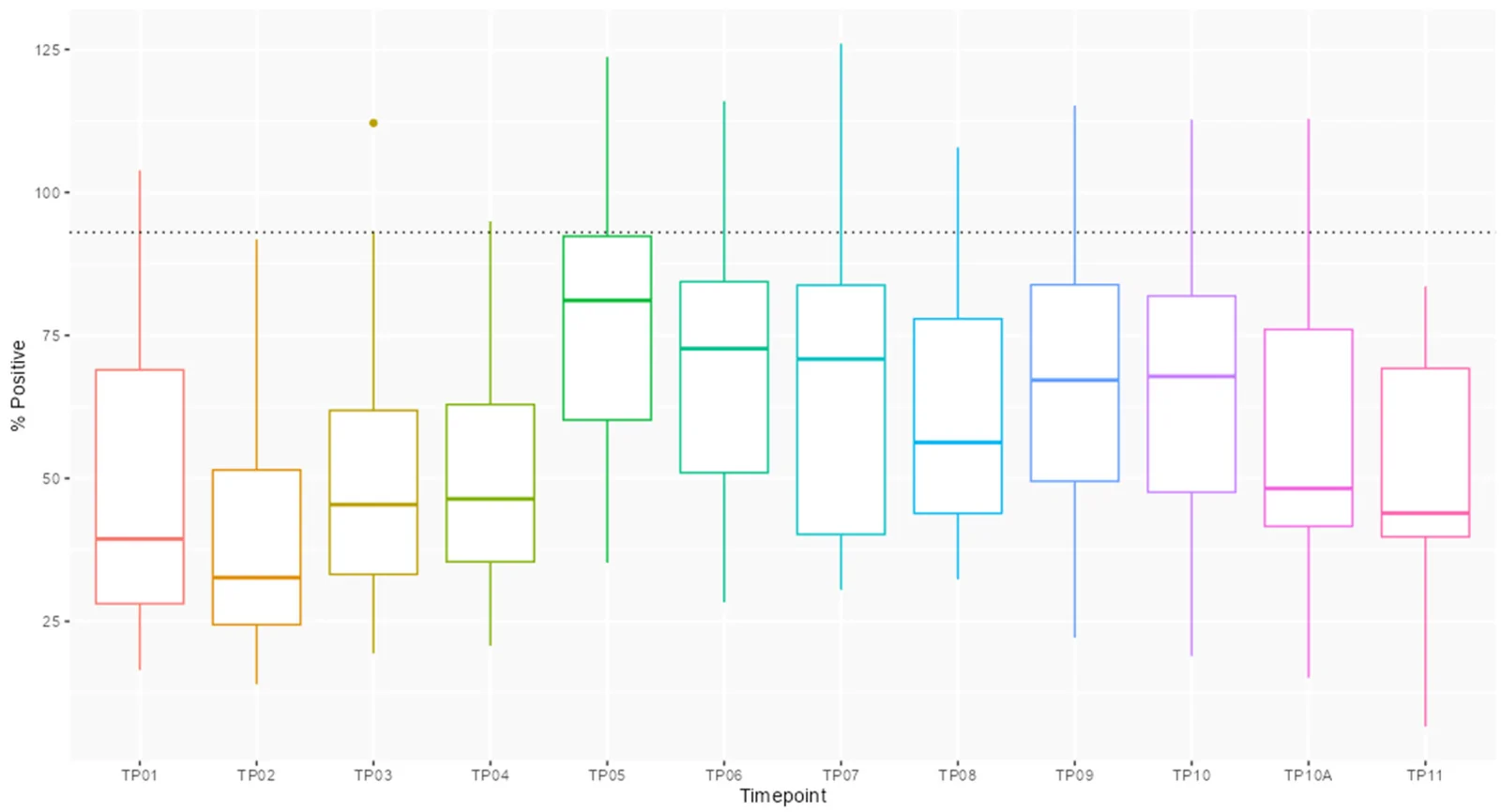
Box-and-whisker plot of responses of thirty animals in the lipid assay with SMP70 as the antigen at each time point. The increase in median from TP4 (bleed 4) to TP5 and TP6 is highly significant, as is the drop from TP5 to TP6, though from TP6 on there are no significant differences. The dotted line shows the cut-off for a positive assignment of MAP infection using antigen SMP70, based on earlier work [53].

The antigens ST123, SMP70 and ZAM295 also showed significant increases in mean from TP4 to TP5 (Figure 4 and Figure 5 for ST123 and SMP70, respectively) (*p* < 0.005). In these cases, however, the reduction in mean following TP6 was not statistically significant (Tables S4a,b, S5a,b and S6a,b).

In the case of ZAM295, the responses were again significantly higher at TP5, but there were also very high responses in some animals at TP1, and the high signals were often maintained after TP5 (Table S6a,b). The responses for JRRR121 were very low for this set of animals relative to a MAP-positive standard, but nonetheless showed statistically significant increases from TP4 to TP5 and TP6, followed by a decrease in median responses (Table S7b).

### 3.4. Comparison with Responses in Serum ID Screen^®^ Assay

**Figure 6:**
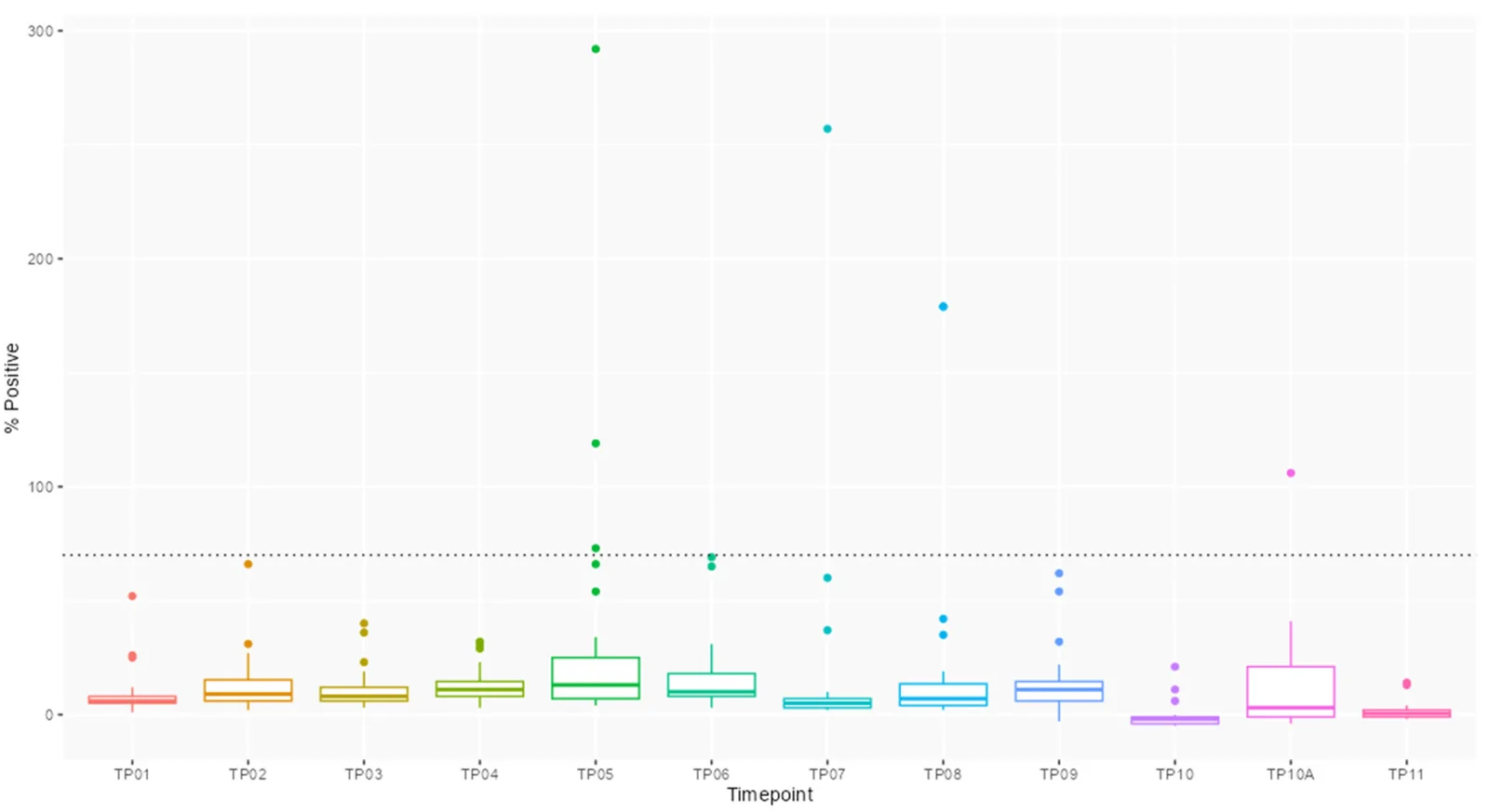
Box-and-whisker plot of responses of thirty animals in the serum ID Screen^®^ assay at each time point (TP). The lines drawn indicate the recommended threshold for a positive result (≥70).

By comparison, the responses for the serum ID Screen^®^ assay are shown in Figure 6.

The data are dominated by a small number of strongly positive signals, which explains the significant differences between the mean and median values (Table S8a). There were no significant increases in the responses for TP1, TP2 and TP3 at any time point up to TP8. Kruskal–Wallis analysis showed a significant increase in responses from TP4 to TP5 (*p* < 0.05) and then a drop to TP6 (*p* < 0.05) (Table S8b).

### 3.5. Comparison with Responses in IGRAs

**Figure 7:**
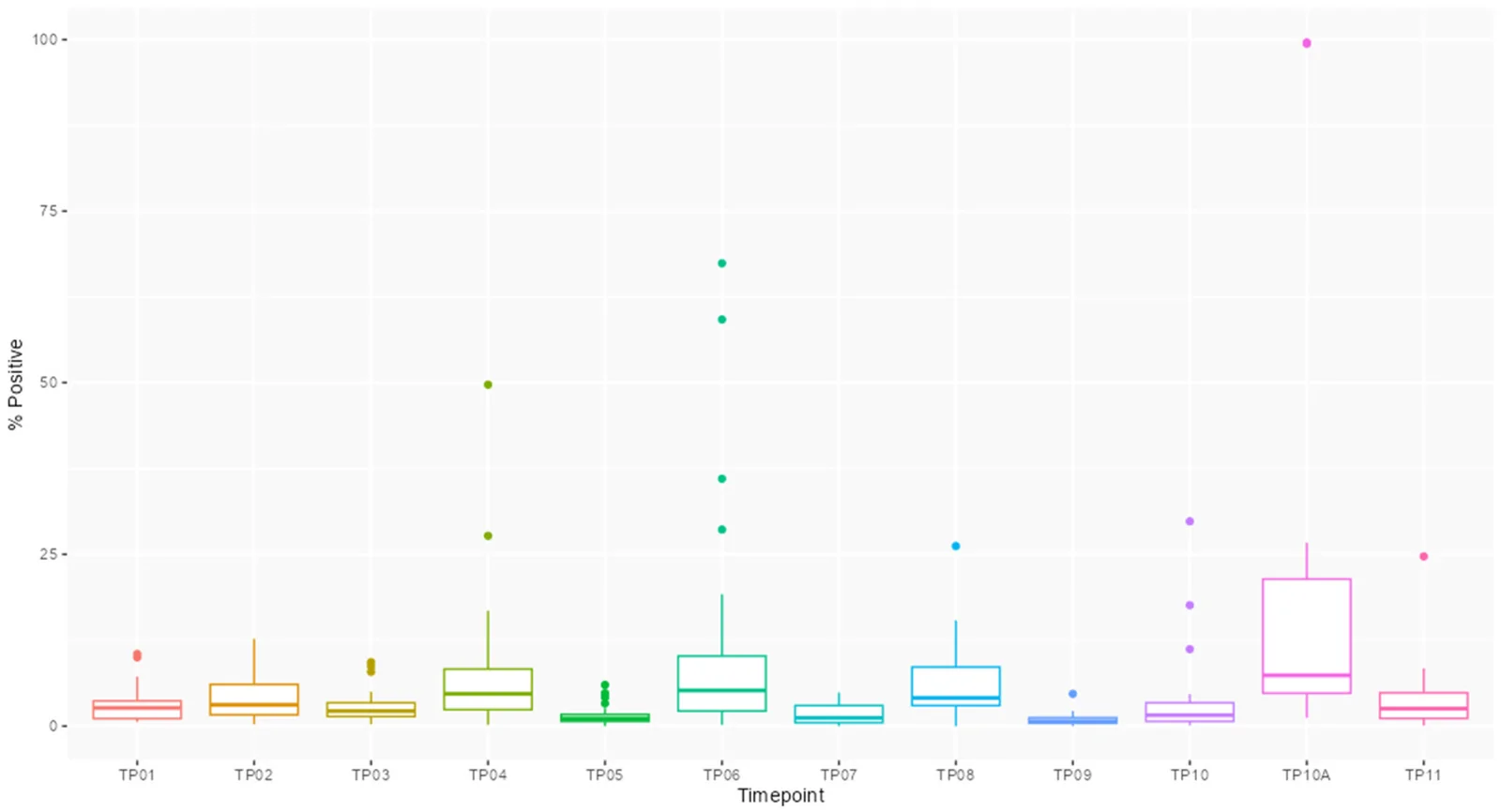
Box-and-whisker plot of responses of thirty animals in the PPDA IGRA at each time point. The drop in median from TP4 to TP5 is highly significant, as is the increase from TP5 to TP6.

The responses in the PPDA IGRA P (Figure 7) and MAP_3651c IGRA, together with an analysis of the changes over time, are given in Tables S9a,b and S10a,b. Like the lipid ELISA test, there is a significant difference between the responses at TP4 and TP5 for both antigens; however, in contrast to the lipid ELISAs, the IGRA responses are lower at time point 5.

The core study on which these results are based [40] involved not only sampling at a series of time points over 5 years, but also the fact that the animals were grazed outside in the summer and housed in the winter. Thus, differences between odd-numbered summer bleeds, when animals were on pasture, and even-numbered bleeds from winter, when housed in sheds, may be seasonal, as previously reported [40].

In order to determine whether this effect could also be important with the six lipid antigens, the total responses for odd and even bleeds were compared for each assay. For the PPDA IGRA, the summer responses were significantly lower than winter responses (*p* < 6 × 10^−7^) (Figure S2.1); the same was true for the MAP_3651c IGRA (Figure S2.2). For the serum ID Screen^®^ assay (Figure S2.3), the reverse effect was observed, with overall winter responses lower than in summer, but the difference was not statistically significant. The medians for each of the lipid antigens (Figures S2.4–S2.9) were also higher in the summer, but the only significant differences were with antigens ST123 and ZAM295 (*p* < 0.005).

### 3.6. Comparison of Results in All Assays

**Figure 8:**
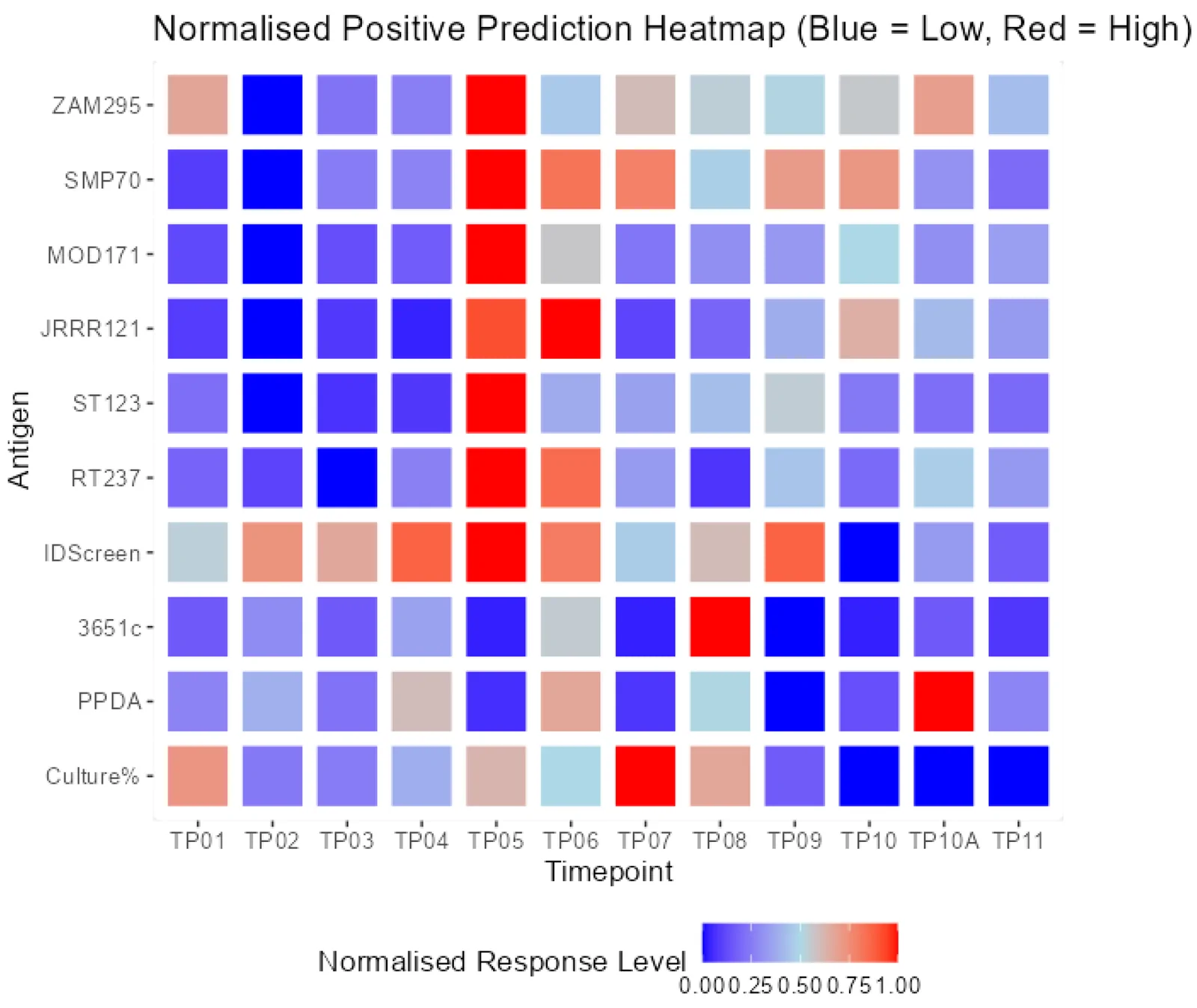
Colour representation of changes in median response in each assay over time. Each assay is separately normalised. For the culture results, each positive at a particular time is counted as 1, and each negative is counted as 0. The corresponding representation of mean responses is shown in Supplementary Figure S3. The responses after TP7 must be evaluated with care because of the removal of some animals for farm management reasons.

A heat map showing the changes in median value over time for the six lipid antigens, ID Screen^®^, culture and the two IGRAs is given in Figure 8.

The correlation between positive responses to antigens MOD171 and RT237F2 at TP5 or TP6 and positive responses at any time in culture or ID Screen^®^ from the published study [40] is given in Table 1 (animal numbers as in column 3 of Table S1), together with the reported responses for those assays at TP5 or TP6. Only one animal (12; Table S1.12) had a positive response in culture at any time but was not detected above the cutoff value at TP5 or TP6 with either of these lipid antigens. In contrast, only three of those animals have a positive ID Screen^®^ response at any time.

Within these data, four of the five samples that were only positive by culture at TP1 and never by ID Screen^®^ were positive by MOD171 or RT237F2 at TP5 or TP6, and in many cases with other antigens. The fifth was positive with SMP70 at later time points. Moreover, another animal that was only positive by culture at TP2 was positive for RT237F2 at TP6. None of these animals were positive in the lipid assays before TP5.

## 4. Discussion

JD is widespread in populations of domesticated ruminants, and there is a potential link to Crohn’s disease in humans. Not surprisingly, there are efforts from the cattle sector and milk processors to reduce the burden of MAP infection in cattle herds [2]. However, the combination of a disease that develops very slowly with ante-mortem diagnostic methods recognised to work effectively only in its later stages of progression is challenging. One of the key diagnostic approaches, detection of the organism in faecal material by PCR or culture, is complicated by intermittent shedding and the possibility of passive shedding. The other commonly used method, an ELISA, normally using purified protein derivative of MAP, a complex mix, primarily of proteins precipitated from cultured, heat-treated MAP, has a very high specificity, but correspondingly a rather low sensitivity. Moreover, it is widely accepted that disease progression is first controlled by Th1 immune responses, and that Th2 responses and high antibody levels can typically take 3–4 years to develop—indeed, some JD control programmes only recommend using current commercial serology tests with animals of this age [52].

The animals in this study were from a highly infected herd and could be considered to have been exposed to MAP early in their lives [40]. Sixteen out of the 30 animals in this study were positive by faecal culture (confirmed by PCR [40]) at least once during the study period, but there were only 35 culture-positive results across the whole cohort. The inconsistencies of the two established diagnostics for MAP are clear, not only in their individual ability to diagnose the disease at all stages, but also in that their results did not always agree. Thus, although the 16 animals were positive by faecal culture (supported by PCR) at some time, only six had a positive ID Screen^®^ serum ELISA score at any time (a total of six positive responses across the cohort), and only three had positive results in both assays. Four of the ID Screen^®^ responses came at TP5, when the animals were three and a half years old, but none were seen before that.

The lipids used in this study were selected from six different classes of MA or ME in order to determine whether there were differences in the patterns of response over the study period. The average responses in the lipid assay for each antigen increased from TP4 to TP5 and TP6. In the case of MOD171 and RT237F2, the response for every single animal showed this increase. In addition, the highest responders to these antigens at either TP5 or TP6, which had lipid ELISA scores above the threshold for determining a positive animal [53] (Table 1), frequently had a positive faecal culture result within the study period—12 and 15 out of the 16 faecal shedding animals respectively were identified with above-threshold responses, and this included five animals that were culture positive only at TP1. The increased responses to antigens MOD171 and RT237F2 at TP5 for every animal in the study herd (albeit in a modest sample size) are worthy of note. Given that, in the UK at least, JD control programmes test beef cow herds every year, inclusion of a MOD171/RT237F2 lipid ELISA at years 3 and 4 might provide a reliable indication of MAP exposure and offer the ability to identify infectious animals prone to shedding. Conversely, the lack of a response at this age may indicate that an animal has not been infected with MAP.

The responses to the lipid antigens have characteristic differences. For example, although MOD171 responses increased from TP4 to TP5 and TP6, those high responses were generally not maintained past this time, unlike the responses to RT237F2, SMP 70 and ST123, which continued to be maintained during the later time points. There are also some inconsistencies in individual animal responses, notably those to antigen ST123, which generally showed good sensitivity and specificity in detecting animals positive by both serum ELISA and PCR [53], but occasionally failed to detect an animal clearly diagnosed by the established tests. The reason for this is unclear but may stem from the inability of some animals to respond to a particular antigen or to changes in response as disease progresses. Thus, the inclusion of more than one antigen in any developed test may be prudent.

We postulate that the observed increases in antibody from TP4 to TP5/TP6 are the result of elevation of Th2 responses, although the immunological data required to support this was not collected during this study. The responses in the two IGRAs, as already reported [40], do fall between TP4 and TP5, as would be predicted by the Th1 to Th2 switch hypothesis; however, this is more likely to be a result of seasonal differences in response, as the IGRA response is recovered at subsequent time points and shows that Th1 and Th2 responses can occur alongside each other. The data presented suggests the timing of the elevation of the Th2 and humoral response to lipid antigens in MAP-infected animals. 

Clearly, this work was limited by the number of infected animals in the longitudinal study and also by the lack of a group from a herd that was believed to be free from MAP. Retention of animals within the herd was solely in the control of the farm manager, whose policy was to remove those with positive test results in the established MAP-based ELISA or because of fertility issues. This inevitably caused attrition bias in the data, particularly after TP7. Studies to address these issues and to establish the test’s performance across different herds are ongoing. Despite the limitations of this study, important conclusions have been drawn, and if the findings of this study are upheld by further research, this test offers the opportunity to identify infected animals before they become infectious. The present study shows that the MA-based assay can provide useful information about the dynamics of the immune response to MAP, can identify individual animals with exposure or infection ahead of the current commercial MAP ELISAs, and possibly indicate high-risk herds.

## 5. Conclusions

Over the whole sample set, there is a wide variety of responses for single samples between culture, ID Screen^®^, IGRA and individual lipid ELISAs. This is not uncommon when trying to diagnose JD and shows just how difficult it can be to decide upon a firm diagnosis of infection, especially before the clinical stage of the disease has been reached. Within this rather complicated picture, the increase in response for every animal in the lipid assay using MOD171 or RT237F2 from bleed TP4 to TP5 and TP6 is striking. This is mirrored in statistically significant increases at this time with several other lipid antigens. Given that all animals in the study were 10–12 months old at the start, one possible explanation is that, in this infected herd, Th2 responses become dominant over Th1 at the age of 3–4 years. Such an explanation would require immunological confirmation. Whatever the explanation, should this consistent response in all exposed animals be confirmed in a much larger study, a positive age-dependent response could provide a reliable serological indicator of exposure. Perhaps more importantly, the lack of a response may be clear evidence of a lack of exposure.

## Figures and Tables

**Table 1:** Comparison of results with MOD171 and RT237F2 at TP5 and TP6 with those from culture and ID Screen^®^ at any time and at TP5/TP6. Cut-offs for +/− for MOD171 and RT237F2 derived from ref. [53].

Animal Number 1–30 as per Supplementary Tables S1.1–S1.30	Predicted +/− at TP5 or TP6 by MOD171	Predicted +/− at TP5 or TP6 by RT237F2	Number of Positive Cultures Any Time by Animal Number	Positive Culture TP5 or TP6	Number of ID Screen^®^ Positives Any Time (≥70% pos. Control)	Positive ID Screen^®^ TP5 or TP6
1,3,15,19	All +	All +	7,4,4,2	All +	0,1,0,0	All −
2,4,5,6,7,8,9,21	All +	All +	2,4,1,1,1,1,1,1	All −	0,2,0,0,0,0,0,0 *	All −
23,27,28	All −	All+	1,1,3	All −	0,0,1	0,0,1
12	Negative	Negative	1	Negative	0	Negative
11,14,18,24,30	All +	All +	0,0,0,0,0	All −	0,0,1,0,0	0,0,1,0,0
13,10,16,17,26,29	All −	All +	0,0,0,0,0,0	All −	0,0,1,0,0,0	All −
20,22,25	All −	All −	0,0,0	All −	0,0 **,1	0,0,1

* One result very close to cut-off. ** Removed after TP2. One positive at TP1B, just after TP1. The cut offs used for the lipid antigens were derived from those used earlier [53].

## Data Availability

The original contributions presented in this study are included in the article/Supplementary Material. Further inquiries can be directed to the corresponding author.

## Supplementary Materials

The following supporting information can be downloaded at https://www.mdpi.com/article/10.3390/ani16162611/s1: Supplementary S1: Bovine tuberculosis testing; Supplementary S2: Timepoints (TP); Figure S1: Structures of synthetic antigens used in this work; Table S1: Data for each animal (S1.1–S1.30 for animals 1–30 (number in first row, third column of each table)) for culture (positive (1) or negative (0), for ID Screen^®^ (recommended cut-off for positive ≥70), for PPDA and 3651c IGRA (as per Hughes et al. [40]), and for six lipid assays (responses as a percentage of a pooled control positive for MAP). Values above recommended cut-offs (derived from Mason et al. [53]) are shown in pink. Animals marked in yellow in the first column have at least two positive culture responses; Table S2: MOD171; Table S2a: Heat-map analysis of results with MOD171, showing an increase in response for every animal between time-points 5 and 6. Animals marked in yellow in column 1 are those with two or more positive culture results; Table S2b: Kruskal—Wallace analysis of data for MOD171; Table S2c: Differences between samples with no positive ID Screen^®^ or culture, those with positive ID Screen^®^ only at TP1, and those with a positive ID Screen^®^ or culture at any other time; Table S3: RT237F2; Table S3a: Heat-map analysis of results with RT237F2. Animals marked in yellow in column 1 are those with two or more positive culture results; Table S3b: Kruskal Wallace analysis of data with RT237F2; Table S4: ST123; Table S4a: Heat-map analysis of results with ST123. Animals marked in yellow in column 1 are those with two or more positive culture results; Table S4b: Kruskal Wallace analysis of data with ST123; Table S5: SMP70; Table S5a: Heat-map analysis of results with SMP70. Animals marked in yellow in column 1 are those with two or more positive culture results; Table S5b: Kruskal Wallis analysis of data with SMP70; Table S6: ZAM295; Table S6a: Heat-map analysis of results with ZAM295. Animals marked in yellow in column 1 are those with two or more positive culture results; Table S6b: Kruskal Wallis analysis of data with ZAM295; Table S7: JJRR121; Table S7a: Heat-map analysis of results with JRRR121. Animals marked in yellow in column 1 are those with two or more positive culture results; Table S7b: Kruskal Wallis analysis of data with JRRR121; Table S8: ID Screen^®^; Table S8a: Reported data for IDScreen^®^ [40]; Table S8b: Kruskal Wallis analysis of data with IDScreen^®^; Table S9: PPDA IGRA; Table S9a: Heat-map analysis of results with PPDA IGRA. Animals marked in yellow in column 1 are those with two or more positive culture results; Table S9b: Kruskal Wallis analysis of data with PPDA IGRA; Table S10: MAP 3651c_IGRA; Table S10a: Heat-map analysis of results with MAP 3651c IGRA. Animals marked in yellow in column 1 are those with two or more positive culture results; Table S10b: Kruskal Wallis analysis of data with MAP 3651c IGRA; Figure S2: Assessment of significant differences between Summer and Winter timepoint data; Figure S2.1: Analysis of Variance (One-Way) for PPDA IGRA data; Figure S2.2: Analysis of Variance (One-Way) for MAP 3651c IGRA data; Figure S2.3: Analysis of Variance (One-Way) for ID Screen^®^ data; Figure S2.4: Analysis of Variance (One-Way) for MOD171 data; Figure S2.5: Analysis of Variance (One-Way) for RT237 data; Figure S2.6: Analysis of Variance (One-Way) for ST123 data; Figure S2.7: Analysis of Variance (One-Way) for JRRR121 data; Figure S2.8: Analysis of Variance (One-Way) for SMP70 data; Figure S2.9: Analysis of Variance (One-Way) for ZAM295 data; Figure S3: Colour representation of changes in mean response in each assay over time. Each assay is separately normalized. For the culture results, each positive at a particular time is counted as 1, and each negative is counted as 0.
