# Supplementary material for: A Longitudinal Study of Mycobacterium avium Subspecies paratuberculosis Infections in Cattle Using Lipid Antigens and Providing Evidence for Age-Associated Antibody Dynamics
Source: Animals (Basel). 2026 Aug 20;16(16):2611. doi: 10.3390/ani16162611 (PMC13509461; doi:10.3390/ani16162611)
Supplement: Supplementary file 1 [file animals-16-02611-s001.zip › animals-4445256-supplementary.pdf]

Supplementary Information

A Longitudinal Study of *Mycobacterium avium* Subspecies *paratuberculosis* Infections in Cattle Using Lipid Antigens and Providing Evidence for Age-Associated Antibody Dynamics

Valerie Hughes, Paul S. Mason, Anna Cervi, Dafydd A. Thomas, George Caldow, Karen Stevenson and Mark S. Baird

S1 Bovine tuberculosis testing: Since September 2009, Scotland has been designated officially tuberculosis-free and has a default routine testing interval of 48 months in the absence of specific risk factors as was the case for the affected, longitudinal study herd. Within the time scale of this trial, the affected herd underwent a statutory single intradermal comparative cervical tuberculin (SICCT) test performed at time point 10. The SICCT test was conducted as follows. Two sites at the caudal margin of the anterior third of the cervical region of each animal were clipped and the skin thickness at each site measured in mm using manual calipers. Animals were injected intradermally at each respective site with 100µl of purified protein derivative of *Mycobacterium avium avium* (PPDA, Thermo Fisher Scientific, Paisley, UK) or 100µl purified protein derivative of Mycobacterium bovis (PPDB, Thermo Fisher Scientific, Paisley, UK) The skin thickness was measured at each site 72 h later. The increase in skin thickness at the PPDB site must exceed that of the PPDA site by more than 4 mm for the animal to be declared a reactor.

S2 Timepoints (TP) refer to separate instances where animals were sampled, samples were generally collected from the animals twice yearly (at approximately six-month intervals) over a period of five years with alternate time points corresponding one to when the animals were at pasture (designated a summer time point), the when the animals were housed (winter time point). Samples were taken at 12 time points in total. In the year in which the SICCT test was performed, an extra sample point was included in order to obtain samples immediately prior to the SICCT test (time point 10) and also one week post SICCT test (time point 10A).

For Timepoints 2, 4, 6, 8, 10, 10A and 11 samples were obtained in winter.

Sixteen animals were culture positive at any point, including 9 at TP7 and seven at TP1. Six animals were above the recommended positive cut-off (70 %) by IDScreen®, two at more than one time. Four animals were positive both by culture and IDScreen®, three at the same time, one at different times. The results for each animal are given in Table S1.

Figure S1: Structures of synthetic antigens used in this work

MOD171

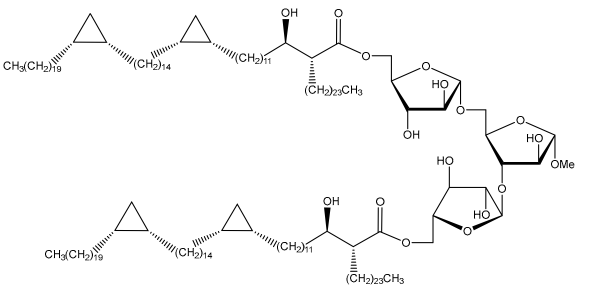

SMP70

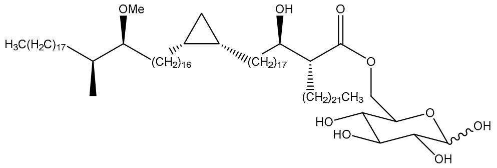

ZAM295

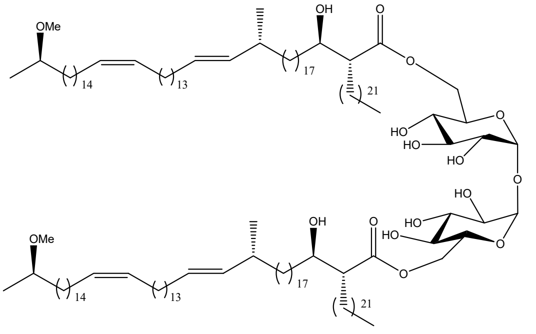

RT237-F2

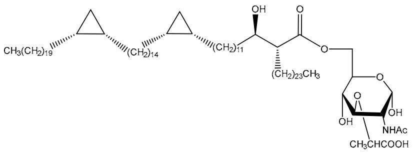

ST123

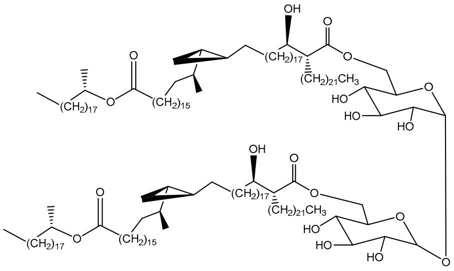

JRRR121

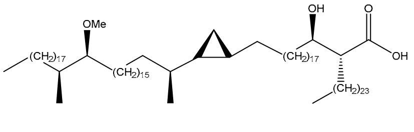

Table S1: Data for each animal (S1.1 – S1.30 for animals 1 – 30 (number in first row, third column of each table)) for culture (positive (1) or negative (0), for ID Screen® (recommended cut-off for positive ≥70), for PPDA and 3651c IGRA (as per Hughes et al [40]), and for six lipid assays (responses as a percentage of a pooled control positive for MAP). Values above recommended cut-offs (derived from Mason et al [53]) are shown in pink. Animals marked in yellow in the first column have at least two positive culture responses.

S1.1		1	TP1	TP2	TP3	TP4	TP5	TP6	TP7	TP8	TP9	TP10	TP10A	TP11
	culture		1	0	1	1	1	1	1	0	1			
MRI316	IDVet		8	19	36	31	20	18	10	9	14			
	3651c		1.7	1.7	0.7	4.7	0.92	14.2	0	5.8	0.8			
	PPDA		4.8	5.3	2.6	12.2	3.3	59.2	1.5	9.7	2.1			
	RT237		40	34	21		70	99	28	15	36			
	ST123		10	15	35		105	51	111	69	73			
	JRRR121		3	-1	1		6	17	-1	0	1			
	MOD171		25	19	46		90	95	17	32	32			
	SMP70		32	37	53		53	55	40	46	115			
	ZAM295		43	57	76		94	77	96	87	88			

S1.2		2	TP1	TP2	TP3	TP4	TP5	TP6	TP7	TP8	TP9	TP10	TP10A	TP11
	culture		0	0	0	0	0	0	1	1	0	0	0	0
MRI324	IDVet		3	6	5	11	12	8	7	11	11	-1	1	2
	3651c		0.7	1.1	0.1	0.5	0.1	0	0.5	0	0	0.3	0.3	0.3
	PPDA		1.1	3.3	0.9	1.8	0	0.2	0.8	0	0.2	0.3	5.2	0.8
	RT237		42	25	24	42	84	104	62	43	50	46	54	
	ST123		47	25	58	48	70	50	63	64	62	54	61	
	JRRR121		1	1	4	7	14	9	2	1	3	2	1	
	MOD171		59	13	43	40	103	160	33	48	42	49	30	
	SMP70		103	92	93	86	85	93	72	77	81	76	73	
	ZAM295		88	88	91	91	92	85	94	92	89	86	89	

S1.3		3	TP1	TP2	TP3	TP4	TP5	TP6	TP7	TP8	TP9	TP10	TP10A	TP11
	culture		0	0	0	0	1	1	1	1				
MRI350	IDVet		2	9	3	3	5	4	10	179				
	3651c		0.3	1.7	0.8	1	0.8	1.3	0	4.9				
	PPDA		1	6.2	3	4.2	6	7	1.4	11.4				
	RT237		29	21	14	33	78	71	45	27				
	ST123		9	6	7	6	10	11	13	17				
	JRRR121		0	-1	-2	-1	5	2	-1	1				
	MOD171		28	8	13	67	145	116	95	85				
	SMP70		16	14	19	24	42	38	30	39				
	ZAM295		32	29	43	30	41	37	41	41				

S1.4		4	TP1	TP2	TP3	TP4	TP5	TP6	TP7	TP8	TP9	TP10	TP10A	TP11
	culture		0	0	1	1	0	0	1	1				
	IDVet		7	27	10	29	54	69	257	179				
	3651c		0	0	0.3	1.2	0.5	0.2	0.4	3				
	PPDA		0.6	4.3	1.8	4.8	2	4.5	1.2	5.2				
	RT237		78	47	23	29	121	93	31	32				
MRI361	ST123		12	65	60	15	27	19	23	27				
	JRRR121		8	4	0	0	7	9	1	6				
	MOD171		60	32	28	7	127	68	16	48				
	SMP70		19	44	36	33	93	101	85	50				
	ZAM295		53	83	85	50	71	55	62	61				

S1.5		5	TP1	TP2	TP3	TP4	TP5	TP6	TP7	TP8	TP9	TP10	TP10A	TP11
	culture		0	0	0	0	0	0	1	0	0	0	0	0
	IDVet		7	8	8	7	11	12	5	10	14	0	17	
	3651c		0.3	1.2	0.4	0	0.2	0	0	3	0.2	0.2	0.9	0.7
	PPDA		4.5	6.4	0.7	5.2	1.5	5.5	2.7	13.2	0.5	4.3	24.9	2.5
	RT237		63	33	34	41	66	98	47	48	80	61		
MRI329	ST123		89	86	50	36	84	68	52	81	78	66		
	JRRR121		6	1	1	3	7	8	3	11	10	10	3	
	MOD171		46	21	31	26	78	115	30	77	77	96	41	
	SMP70		69	53	70	61	82	94	71	80	76	83	78	
	ZAM295		95	95	78	77	98	82	81	91	95	88	88	

S1.6		6	TP1	TP2	TP3	TP4	TP5	TP6	TP7	TP8	TP9	TP10	TP10A	TP11
	culture		1	0	0	0	0	0	0	0	0	0	0	0
	IDVet		11	8	6	7	7	9	6	5	11	-3	21	-1
	3651c		0.5	1.3	1	0.2	0.6	1.4	0.3	1.1	0	0.2	1.2	0.1
	PPDA		1.9	4.4	3.6	10.7	0.9	4.5	1.2	4.1	0.7	0.5	6.2	0.1
	RT237		44		17	33	79	74	28	12	50	27	47	33
MRI334	ST123		23		10	23	30	15	8	9	16	13	12	11
	JRRR121		2		1	1	3	2	-1	-1	9	2	0	0
	MOD171		20		18	23	84	85	99	24	73	59	23	28
	SMP70		63		62	80	91	105	100	72	84	75	73	84
	ZAM295		58		56	71	82	60	71	57	62	61	75	68

S1.7		7	TP1	TP2	TP3	TP4	TP5	TP6	TP7	TP8	TP9	TP10	TP10A	TP11
	culture		1	0	0	0	0	0	0	0	0	0	0	0
MRI352	IDVet		7	20	18	16	6	7	3	9	17	-4	-1	
	3651c		0	0.1	1.7	0.5	0	1.1	1.5	10.8	0	1	0.1	0.2
	PPDA		6.5	5.7	8.7	11.5	4.8	67.4	4.9	26.2	1.2	17.6	26.7	6.5
	RT237		29	28	20	37	71	80	38	21	84	29	43	38
	ST123		2	2	2	4	12	7	7	25	10	8	10	10
	JRRR121		2	0	1	5	1	2	1	5	6	3	2	5
	MOD171		18	15	22	24	71	95	27	30	159	64	35	37
	SMP70		38	49	58	54	81	75	94	78	84	74	71	64
	ZAM295		17	23	25	24	61	38	64	72	50	49	57	59

S1.8		8	TP1	TP2	TP3	TP4	TP5	TP6	TP7	TP8	TP9	TP10	TP10A	TP11
	culture		0	0	0	0	0	0	1	0	0	0	0	0
MRI344	IDVet		6	16	40	22	16	8	3	3	3	-5	0	-2
	3651c		0.1	0.4	0	0.2	0	0	0	0	0	0.1	0.2	0.2
	PPDA		1.1	1.6	1	2.4	0.1	1.6	0.5	3.3	1	1.6	3.6	0.9
	RT237		57	55	42	54	113	112	45	31	54	46	58	58
	ST123		3	4	5	4	36	12	10	13	7	11	12	5
	JRRR121		18	15	27	19	35	43	17	21	20	24	16	18
	MOD171		40	16	18	21	75	69	41	30	33	43	33	40
	SMP70		54	34	45	41	60	73	58	44	55	57	47	45
	ZAM295		77	62	23	32	102	83	77	68	42	72	75	35

S1.9		9	TP1	TP2	TP3	TP4	TP5	TP6	TP7	TP8	TP9	TP10	TP10A	TP11
	culture		1	0	0	0	0	0	0	0	0	0	0	0
MRI341	IDVet		12	7	8	8	14	14	5	4	6	6	-4	-1
	3651c		0.8	2.32	0.6	0.7	0	1.1	0	0.57	0.2	0.2	0.7	0.3
	PPDA		1.9	3.9	2	4.8	0	4.9	0.5	2.4	0.7	0.7	26.4	2.6
	RT237		42	33	29	54	111	67	53	63	37	50	53	40
	ST123		15	5	7	6	33	14	13	14	15	12	9	11
	JRRR121		21	7	15	16	37	18	13	25	3	22	12	9
	MOD171		15	10	17	21	64	24	17	29	23	34	18	12
	SMP70		28	24	33	48	77	43	45	63	30	61	48	43
	ZAM295		73	16	20	22	75	52	49	32	65	34	39	48

S1.10		10	TP1	TP2	TP3	TP4	TP5	TP6	TP7	TP8	TP9	TP10	TP10A	TP11
	culture		0	0	0	0	0	0	0	0	0	0	0	0
MRI321	IDVet		6	6	8	32	7	9	2	2	12	-4	29	-2
	3651c		0	0	0	1.1	0	0.3	0.4	0	0.3	0	0.1	0.1
	PPDA		0.7	0.3	1.8	3.3	1.1	2.2	2.6	2.8	0.6	0.7	4.1	1.7
	RT237		33	22	14	34	81	66	26	13	37	29	39	
	ST123		15	9	7	8	26	10	19	20	122	116	119	
	JRRR121		11	10	17	10	23	18	7	11	14	16	9	
	MOD171		15	11	10	9	29	19	12	10	29	33	31	
	SMP70		75	60	70	64	84	78	80	75	113	113	113	
	ZAM295		82	52	37	47	76	41	69	56	102	105	104	

S1.11		11	TP1	TP2	TP3	TP4	TP5	TP6	TP7	TP8	TP9	TP10	TP10A	TP11
	culture		0	0	0	0	0	0	0	0	0	0	0	0
	IDVet		3	9	12	10	4	9	3	2	5	-4	-1	-2
	3651c		0.8	1.1	1	1.4	0	0.5	0	0	0.2	0.6	2	1.7
	PPDA		3.7	2.5	1	4.6	0.7	3	0.5	1.3	0.7	1.6	21.4	5.3
	RT237		45	39	39	42	91	124	57	32	54	43	62	
MRI335	ST123		3	5	4	6	10	17	5	2	1	3	3	
	JRRR121		17	11	15	11	31	44	19	17	15	42	19	
	MOD171		17	15	15	15	53	66	25	17	22	29	18	
	SMP70		76	38	54	62	67	84	84	46	48	49	43	
	ZAM295		37	29	26	51	56	37	58	34	44	41	28	

S1.12		12	TP1	TP2	TP3	TP4	TP5	TP6	TP7	TP8	TP9	TP10	TP10A	TP11
	culture		1	0	0	0	0	0	0	0	0	0	0	0
	IDVet		8	6	23	11	10	8	4	16	11	-1	8	1
	3651c		0.1	1.2	0.5	0.7	0.3	0.3	1	2	0.3	0.3	1.5	0.9
	PPDA		3.1	6.7	2	1.8	1.7	1.3	4.6	3.8	1.3	4.7	12	3.9
	RT237		37	35	33	41	72	74	39	29	39	37	53	40
MRI347	ST123		28	11	11	14	31	15	29	56	26	39	51	33
	JRRR121		15	11	20	14	15	20	9	18	23	15	9	13
	MOD171		15	9	17	20	32	28	17	24	21	27	24	24
	SMP70		92	46	54	73	81	69	79	101	80	93	99	80
	ZAM295		79	33	32	45	67	32	65	78	54	67	73	55

S1.13		13	TP1	TP2	TP3	TP4	TP5	TP6	TP7	TP8	TP9	TP10	TP10A	TP11
	culture		0	0	0	0	0	0	0	0	0	0	0	0
	IDVet		4	6	7		5	3	3	4	8	-2	3	4
	3651c		0.4	1.09	1.6		0.5	2.9	0	1.1	0.3	1.1	0.2	1.1
	PPDA		2.8	2.5	3		1.6	10.2	2.2	3.2	1.7	3	7.4	4.3
	RT237		32	37	40		127	72	48	35	36	58	67	58
MRI353	ST123		7	3	3		9	4	3	4	3	5	3	3
	JRRR121		9	6	14		31	22	10	12	12	23	14	12
	MOD171		10	7	10		36	29	12	15	23	33	27	30
	SMP70		59	71	93		103	78	95	82	87	68	79	61
	ZAM295		101	73	76	52	76	73	68	95	69	64	62	67

S1.14		14	TP1	TP2	TP3	TP4	TP5	TP6	TP7	TP8	TP9	TP10	TP10A	TP11
	culture		0	0	0	0	0	0	0	0	0	0	0	0
	IDVet		6	10	13	12	6	8	5	4	15	-4	8	0
	3651c		0	0	0.3	0.7	0	0	0	0.6	0.1	0	0.3	0.1
	PPDA		0.7	2.3	2.9	1.6	0.8	2	0	2.6	0.6	0.1	5.4	1.3
	RT237		36	38	38	40	84	70	58	41	62	251	256	70
MRI346	ST123		90	41	28	21	50	55	38	70	38	28	24	29
	JRRR121		14	8	21	9	31	19	10	17	14	25	14	9
	MOD171		92	69	48	31	68	74	55	39	41	34	30	27
	SMP70		19	20	26	21	35	28	34	80	47	48	42	39
	ZAM295		101	73	76	52	76	73	68	95	69	64	62	67

S1.15		15	TP1	TP2	TP3	TP4	TP5	TP6	TP7	TP8	TP9	TP10	TP10A	TP11
	culture		0	0	0	0	1	1	1	1	0			
MRI348	IDVet		5	5	5	6	25	10	5	35	32			
	3651c		0	0	0.4	0	0	1.7	0.2	1.7	1.1			
	PPDA		0.6	1.5	2.3	0.9	0.2	19.2	0	3.8	1.2			
	RT237		35	16	16	32	86	61	52	35	52			
	ST123		17	3	4	5	76	27	16	19	22			
	JRRR121		12	6	8	6	23	18	13	12	14			
	MOD171		11	5	6	10	44	27	14	11	26			
	SMP70		43	27	47	41	105	91	86	86	80			
	ZAM295		46	12	20	32	90	47	49	42	34			

S1.16		16	TP1	TP2	TP3	TP4	TP5	TP6	TP7	TP8	TP9	TP10	TP10A	TP11
	culture		0	0	0	0	0	0	0	0	0	0	0	0
MRI333	IDVet		26	20	19	13	23	18	9	19	62	11	106	13
	3651c		0.9	0.2	0	0.3	0	2.1	0.7	0	0	0	0	1.1
	PPDA		2.8	2.9	2.2	1.7	1.3	10.1	4.6	3.9	0.6	0.9	4.8	4.7
	RT237		47	30	12	30	97	87	23	13	97	36	57	58
	ST123		7	7	10	8	37	6	13	18	78	25	25	22
	JRRR121		15	3	6	2	21	28	8	6	30	19	13	8
	MOD171		27	18	29	19	50	44	25	26	52	36	28	26
	SMP70		104	74	79	69	92	84	82	66	98	82	76	73
	ZAM295		95	84	89	75	94	61	87	91	104	95	98	96

S1.17		17	TP1	TP2	TP3	TP4	TP5	TP6	TP7	TP8	TP9	TP10	TP10A	TP11
	culture		0	0	0	0	0	0	0	0	0	0	0	0
MRI317	IDVet		10	12	7	9	14	11	7	8	9	-1	22	0
	3651c		0.3	0	0	1.5	0.2	0.6	0	1.3	0.2	0.1	0.4	0.1
	PPDA		2.5	0.8	1.4	6.3	0.5	7.7	0	5	0.9	1.6	2.3	1.8
	RT237		24	33	22	30	84	44	88	18	110	33	49	
	ST123		10	3	25	37	37	14	36	47	55	26	45	
	JRRR121		4	2	3	4	13	8	9	7	17	9	7	
	MOD171		23	13	18	17	39	25	25	25	54	21	20	
	SMP70		93	51	54	65	60	51	51	42	75	50	42	
	ZAM295		95	72	88	99	94	94	95	97	95	94	96	

S1.18		18	TP1	TP2	TP3	TP4	TP5	TP6	TP7	TP8	TP9	TP10	TP10A	TP11
	culture		0	0	0	0	0	0	0	0				
MRI332	IDVet		6	31	16	12	73	17	4	6				
	3651c		0.8	0	0.1	0	0	0.4	0.2	0				
	PPDA		1.1	0.5	0.3	0.2	0.2	1.6	0.9	0				
	RT237		28	22	17	25	123	76	52	18				
	ST123		12	8	3	18	148	25	23	29				
	JRRR121		6	20	12	6	33	8	4	2				
	MOD171		12	12	15	12	76	27	21	16				
	SMP70		31	27	26	29	82	40	38	32				
	ZAM295		79	59	26	62	101	69	82	78				

S1.19		19	TP1	TP2	TP3	TP4	TP5	TP6	TP7	TP8	TP9	TP10	TP10A	TP11
	culture		0	0	0	0	1	0	1	0	0	0	0	
	IDVet		5	8	5	11	6	6	6	7	-3	-1	-1	
	3651c		0.6	1.6	1.8	3.3	0.4	6.2	0.3	4.1	0.1	2.4	3.6	
	PPDA		2.5	3.7	5	16.8	0.9	6.4	0.9	8.8	0.3	29.8	6.3	
	RT237		31	20	43	50	102	66	42	20	39	35	54	
MRI331	ST123		14	2	7	4	115	20	48	16	13	12	12	
	JRRR121		9	3	5	4	14	8	4	3	7	4	4	
	MOD171		13	9	11	18	57	31	22	16	19	23	16	
	SMP70		23	18	33	35	97	65	85	48	51	45	46	
	ZAM295		73	14	62	31	100	82	95	74	76	66	66	

S1.20		20	TP1	TP2	TP3	TP4	TP5	TP6	TP7	TP8	TP9	TP10	TP10A	TP11
	culture		0	0	0	0	0	0	0	0	0	0	0	0
	IDVet		1	5	6	3	26	22	37	18		-1	3	3
	3651c		0.2	2.9	1.2	5.3	1.13	6.9	0.1	3.5	0	0.1	1.9	0
	PPDA		2.1	12.2	3.7	10.7	1.4	36	0.3	10.4	0	3	9.3	3.9
	RT237		21	23	13	30	71	55	29	11	30	35	43	44
MRI360	ST123		2	2	3	39	54	27	6	9	4	10	11	15
	JRRR121		3	1	4	3	1	0	3	2	4	8	9	4
	MOD171		11	4	15	15	34	34	12	11	14	17	14	10
	SMP70		18	15	35	29	56	45	33	33	30	34	31	35
	ZAM295		24	15	14	73	92	73	33	40	30	35	41	59

S1.21		21	TP1	TP2	TP3	TP4	TP5	TP6	TP7	TP8	TP9	TP10	TP10A	TP11
	culture		1											
	IDVet		5	10	12	23	66	28	7					
	3651c		0.2	0.3	0.2	0	0.7	1.1	1.2					
	PPDA		0.9	0.9	0.4	2.4	0.7	4.3	3.3					
	RT237		33	17	25	36	212	134	59					
MRI340	ST123		13	4	10	5	115	102	79					
	JRRR121		3	5	20	17	38	52	32					
	MOD171		15	10	24	24	97	101	85					
	SMP70		41	30	44	39	109	84	74					
	ZAM295		72	26	48	17	95	97	100					

S1.22		22	TP1	TP2	TP3	TP4	TP5	TP6	TP7	TP8	TP9	TP10	TP10A	TP11
	Culture		0	0										
MRI354	IDVet		52	66			Note 86 at TP1b – within a month of TP1							
	3651c		0.5	0.4										
	PPDA		3.6	2.9										
	RT237		43	35										
	ST123		8	19										
	JRRR121		5	3										
	MOD171		15	11										
	SMP70		68	24										
	ZAM295		45	63										

S1.23		23	TP1	TP2	TP3	TP4	TP5	TP6	TP7	TP8	TP9	TP10	TP10A	TP11
	culture		0	1	0	0	0	0	0	0	0			
MRI323	IDVet		6	8	4	16	7	6	5	4	8			
	3651c		1.5	0	0.8	1.4	0.3	0.8	0	0.2	0.7			
	PPDA		3.3	2.1	2	5.6	1.3	5.2	0.9	3.8	4.7			
	RT237		24	26	27	42	90	76	52	32	71			
	ST123		34	5	10	7	30	10	8	12	23			
	JRRR121		5	3	3	3	11	16	5	5	8			
	MOD171		15	9	11	14	32	18	15	14	19			
	SMP70		41	32	23	47	40	36	39	39	59			
	ZAM295		89	41	69	49	87	52	51	54	59			

S1.24		24	TP1	TP2	TP3	TP4	TP5	TP6	TP7	TP8	TP9	TP10	TP10A	TP11
	culture		0	0	0	0	0	0	0	0	0	0	0	0
MRI358	IDVet		3	9	5	8	7	10	4	4	6	-4	1	-2
	3651c		0	0.8	0.1	0.6	0.6	1.1	0.4	4.5	0.5	0	6.4	3.4
	PPDA		7.2	12.2	4.4	7.5	4.2	16.7	3	15.4	2.2	3.4	99.6	25
	RT237		25	31	15	41	123	104	40	32	55	68	90	76
	ST123		30	5	71	24	88	50	86	70	56	55	59	41
	JRRR121		4	3	4	4	17	15	5	9	8	11	9	9
	MOD171		12	9	17	18	57	29	24	24	29	30	24	29
	SMP70		28	18	112	59	124	98	126	108	95	93	101	71
	ZAM295		80	25	102	59	103	86	105	103	92	95	97	80

S1.25		25	TP1	TP2	TP3	TP4	TP5	TP6	TP7	TP8	TP9	TP10	TP10A	TP11
	culture		0	0	0	0	0	0	0					
MRI318	IDVet		2	12	7	11	292	26	3					
	3651c		0.5	0.9	0.8	1.8	0	1.2	0					
	PPDA		10.5	12.7	7.9	49.7	1.1	28.6	1					
	RT237		60	40	35	40	75	72	22					
	ST123		6	4	6	15	121	17	18					
	JRRR121		5	0	5	3	16	3	-3					
	MOD171		20	11	16	12	49	30	8					
	SMP70		27	33	33	35	68	38	35					
	ZAM295		41	42	51	85	101	55	77					

S1.26		26	TP1	TP2	TP3	TP4	TP5	TP6	TP7	TP8	TP9	TP10	TP10A	TP11
	culture		0	0	0	0	0	0	0	0	0	0	0	0
MRI339	IDVet		25	13	12	14	8	7	3	5	22	-2	1	0
	3651c		0.3	2.5	0.1	0.2	0	0.6	1.2	2.3	0	0.1	0.4	0
	PPDA		2.9	11.1	2.5	3.5	1.3	1.4	3	5.2	0.9	0.3	9.6	1.2
	RT237		31	24	29	39	86	99	47	28	48	25	58	77
	ST123		1	2	2	1	7	7	3	2	8	6	4	4
	JRRR121		-3	-1	0	0	3	7	-1	0	1	2	2	3
	MOD171		8	4	15	6	24	25	13	10	16	14	19	17
	SMP70		30	25	39	40	71	68	53	42	50	82	50	40
	ZAM295		8	4	10	8	25	23	13	13	29	13	15	20

S1.27		27	TP1	TP2	TP3	TP4	TP5	TP6	TP7	TP8	TP9	TP10	TP10A	TP11
	culture		0	0	0	0	0	0	1	0	0	0	0	0
MRI349	IDVet		10	16	11	8	16	8	3	7	12	-2	25	2
	3651c		1.8	0.6	1.2	0	0.4	0	2.3	3.9	0	0.6	1	2.1
	PPDA		10	6.4	9.3	27.7	2.6	7	4.8	8.2	0.2	11.2	18.1	8.4
	RT237		46	30	24	37	82	90	38	42	54	47	52	37
	ST123		2	3	5	1	7	6	2	5	4	4	2	0
	JRRR121		3	5	2	0	3	11	18	7	8	11	10	8
	MOD171		10	7	9	5	24	36	10	15	14	16	8	6
	SMP70		34	58	31	41	54	73	32	49	51	45	35	40
	ZAM295		61	58	52	62	87	38	36	53	37	36	29	19

S1.28		28	TP1	TP2	TP3	TP4	TP5	TP6	TP7	TP8	TP9	TP10	TP10A	TP11
	culture		0	0	0	1	1	1	0	0	0	0	0	0
	IDVet		5	4	7	11	119	65	60	42	54	21	41	14
	3651c		0.4	0.2	0.3	1.2	0.6	2.4	1.4	4.2	0.9	0.3	5.9	1.6
	PPDA		0.7	1.5	3.4	4.2	2.5	10.2	3.4	8.4	1.3	2.1	99.4	7.5
	RT237		47	33	39	46		69	49		47	30	37	37
MRI351	ST123		17	17	24	34		35	100		59	55	59	54
	JRRR121		3	4	15	6		18	12		5	4	3	2
	MOD171		8	10	14	32		96	85		23	18	15	15
	SMP70		25	31	43	46		57	67		55	42	38	43
	ZAM295		98	71	69	102		96	101		96	101	102	102

S1.29		29	TP1	TP2	TP3	TP4	TP5	TP6	TP7	TP8	TP9	TP10	TP10A	TP11
	culture		0	0	0	0	0	0	0	0	0	0	0	0
	IDVet		6	2	5	4	34	31	2	3	5	-5	-2	2
	3651c		1.6	0.6	0.4	9.5	0	1	0.2	4.1	0	0.5	0.3	0.2
	PPDA		4.3	1.5	1.8	5.5	0.9	4.7	0.1	6.3	0.2	2.2	1.2	0.3
	RT237		34	34	19	41	93	92	40	21	58	40	46	72
MRI336	ST123		3	5	3	3	80	8	6	4	4	7	2	2
	JRRR121		7	4	8	8	10	10	4	3	6	13	5	5
	MOD171		6	3	9	6	34	27	4	5	11	33	8	7
	SMP70		37	22	32	26	96	70	55	44	49	71	40	39
	ZAM295		20	31	13	11	102	71	51	56	51	5	14	11

S1.30		30	TP1	TP2	TP3	TP4	TP5	TP6	TP7	TP8	TP9	TP10	TP10A	TP11
	culture		0	0	0	0	0	0	0	0	0	0	0	0
	IDVet		8	6	8	13	13	14	3	8	6	-3	-2	1
	3651c		0.9	0.4	0.3	0.1	0.1	0	0	0	0	0.2	0.2	0.2
	PPDA		3.2	1.8	1	2.8	0.3	0.9	4.39	1.4	0.2	1.2	2.1	0.5
	RT237		16	14	10	54	75	114	36	23	50	31	45	36
MRI338	ST123		3	3	4	6	9	41	8	41	22	32	33	20
	JRRR121		3	4	1	1	8	56	1	2	3	1	3	2
	MOD171		1	0	5	6	36	127	17	19	24	29	24	27
	SMP70		76	63	62	95	80	116	75	75	22	19	15	7
	ZAM295		6	2	16	38	43	80	52	105	84	91	96	79

Table S1: Data for each animal (S1.1 – S1.30) for culture (positive (1) or negative), for ID Screen^R (recommended cut-off for positive ≥70), for PPDA and 3651c IGRAs (as per Hughes et al [40]), and for six lipid assays (responses as a percentage of a pooled control positive for MAP). Values above recommended cut-offs (derived from [53]) are shown in pink

Tables S2-S9: Statistical analyses of means for each assay at each TP

Initial statistical analysis in each case was by Anova followed by Tukey HSD/Kramer analysis. The distribution of results in Anova was analysed in R by generation of a Q-Q plot to determine whether the data follow a normal distribution, which would be shown by a straight line. In fact the data in each case did not follow a straight line distribution, and therefore additional statistical analysis was carried out. Firstly an Anova analysis of ln(data), corrected to ensure no negative data and no 0 data, again with Tukey HSD/Kramer secondary analysis; in this case the Q-Q plots with the six lipid antigens were closer to a linear distribution. Secondly by using the Kruskal

Wallis method. The data for Anova are available if required, but not used. The data points identified as significant lnAnova and for Kruskal Wallis were very similar. Those for Kruskal Wallis are used in the discussion. In each case the results comparing differences between one timepoint and each of the others are shown as a matrix. Where a result is significant (p <0.05) it is shown in green.

Table S2: MOD171

MOD171												
Serum ID	TP1	TP2	TP3	TP4	TP5	TP6	TP7	TP8	TP9	TP10	TP10A	TP11
MRI316	24.82	18.52	45.59		89.75	95.35	17.44	31.76	32.01			
MRI324	58.80	13.01	42.71	40.10	102.67	160.44	32.62	48.33	41.93	48.65	30.20	
MRI350	28.06	7.83	12.83	67.17	145.12	115.96	94.69	85.40				
MRI361	59.95	32.03	27.76	7.26	126.76	67.57	16.29	48.32				
MRI329	46.07	20.96	31.21	26.25	78.33	115.28	29.64	76.51	76.71	96.19	40.92	
MRI334	20.07		17.87	22.97	84.38	84.84	98.81	23.82	73.47	58.96	23.05	27.59
MRI352	18.30	14.65	21.67	24.00	70.88	94.80	26.50	29.94	158.94	64.07	34.68	36.79
MRI344	39.63	16.10	18.00	20.78	75.42	69.30	41.19	30.41	33.01	42.57	33.20	39.76
MRI341	15.42	10.03	16.71	20.68	63.87	24.24	16.99	29.34	23.37	34.00	18.50	11.52
MRI321	14.53	10.89	9.85	8.91	28.93	18.89	12.41	10.00	28.60	32.63	30.88	
MRI335	16.98	14.81	15.17	14.57	53.37	66.03	25.03	17.03	22.01	28.99	18.25	
MRI347	15.45	8.87	17.37	19.67	32.26	27.66	17.02	23.68	20.51	27.49	23.68	23.64
MRI353	9.82	6.51	9.52		36.37	29.11	12.24	14.86	22.52	33.33	26.62	30.35
MRI346	91.98	69.20	47.80	31.42	67.96	74.28	55.43	38.75	41.27	33.73	29.93	26.74
MRI348	11.02	5.31	6.31	10.13	43.88	27.46	14.15	10.71	26.09			
MRI333	26.82	17.64	29.44	19.06	50.29	43.64	24.79	25.85	51.58	36.48	27.51	25.64
MRI317	23.01	12.98	18.40	17.16	39.15	25.26	25.21	24.98	54.32	21.47	20.27	
MRI332	12.50	11.69	15.15	12.16	76.09	27.42	20.58	16.47				
MRI331	12.83	8.75	11.43	17.71	57.25	31.25	22.44	16.36	19.37	23.26	15.96	
MRI360	11.32	3.75	14.91	15.45	33.70	33.75	12.00	11.03	14.02	16.62	14.32	10.49
MRI340	15.37	10.00	24.43	24.04	97.49	100.95	85.21					
MRI323	14.63	8.63	11.20	13.61	31.78	17.96	14.60	14.42	19.30			
MRI358	12.30	8.82	16.78	17.58	57.20	29.28	24.02	24.28	29.02	30.00	23.86	28.60
MRI318	20.43	11.23	15.98	12.25	49.14	29.52	7.93					
MRI339	7.83	3.81	15.40	6.27	23.98	25.24	12.91	10.27	16.36	14.26	18.59	17.28
MRI349	9.97	6.65	8.91	5.13	23.97	35.58	10.38	15.13	14.22	15.98	8.00	5.73
MRI351	7.60	10.14	14.30	32.04		95.75	85.04		23.37	18.24	15.02	15.22
MRI336	5.69	3.07	8.90	5.67	34.31	26.96	3.62	4.66	11.13	33.32	7.82	7.25
MRI338	0.58	0.10	5.41	5.87	36.35	126.87	17.40	19.48	23.72	28.79	24.19	27.43
Comment		Winter		Winter		Winter		Winter		Winter	Post skin test	
Mean	22.476	13.070	19.000	19.182	61.095	59.332	30.226	26.992	36.535	35.192	23.117	22.268
Median	15.418	10.086	15.985	17.583	55.285	35.576	20.575	23.752	24.906	32.631	23.676	25.640
	46.28	34.65	26.60	36.15	84.54	89.20	51.21	45.03	85.03	55.22	24.37	22.74

Table S2a: **Heat-map analysis of results with MOD171, showing an increase in response for every animal between time-points 5 and 6. Animals marked in yellow in column 1 are those with two or more positive culture results**

TP	1	2	3	4	5	6	7	8	9	10	10A
2	0.049										
3	0.874	0.078									
4	0.874	0.083	0.999								
5	0.000	0.000	0.000	0.000							
6	0.000	0.000	0.000	0.000	0.483						
7	0.254	0.001	0.200	0.200	0.000	0.001					
8	0.247	0.001	0.197	0.200	0.000	0.001	0.969				
9	0.019	0.000	0.011	0.012	0.007	0.057	0.231	0.255			
10	0.003	0.000	0.002	0.002	0.065	0.231	0.076	0.090	0.561		
10A	0.254	0.002	0.200	0.200	0.000	0.002	0.952	0.981	0.302	0.119	
11	0.475	0.015	0.370	0.370	0.000	0.002	0.874	0.868	0.231	0.089	0.861

Table S2b: Kruskal—Wallace analysis of data for MOD171

MOD171

	Group	TP4	TP5	TP5/TP4	TP5-TP4
MRI321	0	8.9	28.9	3.2	20.0
MRI335	0	14.6	53.4	3.7	38.8
MRI346	0	31.4	68.0	2.2	36.5
MRI317	0	17.2	39.1	2.3	22.0
MRI360	0	15.5	33.7	2.2	18.2
MRI358	0	17.6	57.2	3.3	39.6
MRI339	0	6.3	24.0	3.8	17.7
MRI336	0	5.7	34.3	6.1	28.6
MRI338	0	5.9	36.3	6.2	30.5
MRI324	1	40.1	102.7	2.6	62.6
MRI350	1	67.2	145.1	2.2	77.9
MRI361	1	7.3	126.8	17.5	119.5
MRI329	1	26.3	78.3	3.0	52.1
MRI344	1	20.8	75.4	3.6	54.6
MRI348	1	10.1	43.9	4.3	33.7
MRI333	1	19.1	50.3	2.6	31.2
MRI332	1	12.2	76.1	6.3	63.9
MRI331	1	17.7	57.3	3.2	39.5
MRI323	1	13.6	31.8	2.3	18.2
MRI318	1	12.3	49.1	4.0	36.9
MRI349	1	5.1	24.0	4.7	18.8
MRI334	2	23.0	84.4	3.7	61.4
MRI352	2	24.0	70.9	3.0	46.9
MRI341	2	20.7	63.9	3.1	43.2
MRI347	2	19.7	32.3	1.6	12.6
MRI340	2	24.0	97.5	4.1	73.5
Groups compared		p adj	p adj	p adj	p adj
G1-G0		0.3	0.0	0.7	0.1
G2-G0		0.5	0.2	0.9	0.3
G2-G1		1.0	1.0	0.6	1.0

0 = never positive on IDScreen or culture
1 = positive in either at any TP other than TP1
2 = positive in culture only at TP1

Table S2c: Differences between samples with no positive ID Screen® or culture, those with positive ID Screen® only at TP1, and those with a positive ID Screen® or culture at any other time

Table S3: RT237F2

RT237F2												
Serum ID	TP1	TP2	TP3	TP4	TP5	TP6	TP7	TP8	TP9	TP10	TP10A	TP11
MRI316	40.22	34.31	21.45		69.98	98.99	27.65	15.31	35.89			
MRI324	42.33	24.50	24.22	41.93	84.31	103.68	61.96	43.29	50.22	46.11	54.36	
MRI350	28.73	21.14	14.17	33.14	77.84	70.66	44.86	27.18				
MRI361	78.43	46.95	22.57	28.76	120.61	92.74	31.28	31.55				
MRI329	63.04	33.28	33.57	40.89	66.19	98.12	47.24	48.22	80.07	60.92		
MRI334	44.38		16.57	33.14	79.16	74.31	28.20	11.59	50.31	26.86	47.21	33.40
MRI352	29.32	28.17	19.61	36.63	71.34	80.03	38.10	21.16	84.46	29.29	42.53	38.40
MRI344	56.64	54.67	41.51	53.55	112.83	112.08	44.51	31.10	53.65	45.75	58.41	58.30
MRI341	41.58	33.18	29.21	54.17	111.43	66.72	53.50	62.98	37.17	49.58	53.30	39.50
MRI321	32.75	22.08	14.28	33.79	80.59	65.55	25.76	12.86	36.81	29.41	39.36	
MRI335	45.03	38.79	39.10	41.91	91.36	124.30	56.68	31.60	54.10	43.46	61.94	
MRI347	36.72	35.43	33.23	41.43	71.84	73.52	39.26	29.01	39.44	36.52	52.76	40.50
MRI353	31.74	36.70	40.37		126.55	72.29	47.54	35.17	36.21	57.56	66.90	58.33
MRI346	35.74	38.13	37.91	40.30	84.26	70.46	57.76	40.75	61.57	251.41	255.99	69.83
MRI348	35.49	15.92	15.80	32.38	86.30	61.18	52.02	34.94	52.41			
MRI333	47.26	29.88	12.09	29.87	96.86	86.70	23.40	13.00	97.28	35.63	56.62	57.83
MRI317	23.66	33.30	22.05	29.67	83.95	44.17	87.79	18.02	110.47	32.64	49.12	
MRI332	28.20	21.91	16.95	24.69	123.36	75.56	51.64	18.12				
MRI331	31.28	20.43	43.36	50.14	102.49	66.36	42.03	20.44	38.50	35.44	53.99	
MRI360	21.44	22.93	12.88	30.39	70.88	55.00	29.13	11.16	29.58	35.30	42.80	44.24
MRI340	32.91	16.91	24.76	35.83	212.38	133.89	59.41					
MRI323	23.77	26.13	26.78	41.68	90.04	75.78	51.87	32.21	70.69			
MRI358	25.05	31.08	15.44	41.42	123.31	103.85	40.28	31.63	54.66	67.76	89.77	76.12
MRI318	59.90	39.56	35.12	40.32	75.14	71.82	22.16					
MRI339	30.88	24.16	28.98	38.82	86.27	98.64	47.19	28.41	47.64	25.05	57.89	77.07
MRI349	46.29	29.96	24.17	36.75	81.97	89.60	37.95	42.09	53.65	47.13	51.55	36.98
MRI351	46.50	33.49	38.77	45.99		68.88	49.24		47.31	30.12	37.35	36.96
MRI336	33.97	34.22	18.80	41.14	93.47	92.22	39.98	20.56	58.24	39.76	45.69	72.23
MRI338	15.68	14.02	10.50	53.90	74.97	113.77	36.18	23.38	50.20	30.72	44.51	36.27
Comment		Winter		Winter		Winter		Winter		Winter	Post skin test	
Mean	38.24	30.04	25.32	38.99	94.63	84.17	43.95	28.30	55.44	50.31	63.10	51.73
Median	35.49	30.52	24.17	40.30	85.29	75.78	44.51	28.71	51.36	36.52	53.03	44.24
Midrange	47.05	34.35	26.93	39.43	139.28	89.03	54.98	37.07	70.03	138.23	146.67	55.23

Table S3a: Heat-map analysis of results with RT237F2. Animals marked in yellow in column 1 are those with two or more positive culture results

TP	1	2	3	4	5	6	7	8	9	10	10A
2	0.0969										
3	0.0134	0.4591									
4	0.6995	0.0409	0.0046								
5	0.0000	0.0000	0.0000	0.0000							
6	0.0000	0.0000	0.0000	0.0000	0.6847						
7	0.2221	0.0039	0.0002	0.4560	0.0000	0.0000					
8	0.0600	0.7818	0.6402	0.0247	0.0000	0.0000	0.0022				
9	0.0059	0.0000	0.0000	0.0203	0.0024	0.0082	0.1145	0.0000			
10	0.5149	0.0263	0.0030	0.7415	0.0000	0.0000	0.6913	0.0153	0.0605		
10A	0.0030	0.0000	0.0000	0.0105	0.0105	0.0309	0.0624	0.0000	0.7347	0.0336	
11	0.0409	0.0007	0.0000	0.0944	0.0028	0.0082	0.3339	0.0003	0.7347	0.1944	0.5630

Table S3b: Kruskal Wallace analysis of data with RT237F2

Table S4: ST123

ST123												
Serum ID	TP1	TP2	TP3	TP4	TP5	TP6	TP7	TP8	TP9	TP10	TP10A	TP11
MRI316	9.69	15.22	34.73		105.08	50.89	111.47	68.57	72.80			
MRI324	46.76	25.11	57.98	48.04	70.20	50.48	62.72	64.19	62.27	53.91	60.91	
MRI350	8.57	5.73	7.32	5.73	10.21	10.91	13.22	16.64				
MRI361	12.35	65.01	60.25	14.67	26.66	19.06	22.84	26.91				
MRI329	89.41	85.66	50.24	36.30	84.13	67.80	52.30	80.79	77.65	66.31		
MRI334	23.07		10.04	23.00	30.41	15.04	7.52	9.49	16.48	12.66	11.80	10.79
MRI352	2.02	2.34	2.00	3.75	12.12	6.71	6.78	25.41	10.09	8.00	9.94	10.43
MRI344	3.44	4.04	4.75	4.14	35.70	12.47	9.89	12.78	6.61	11.49	11.69	4.99
MRI341	15.35	5.48	6.51	5.85	32.90	13.51	12.92	13.51	14.98	12.47	8.74	11.41
MRI321	15.27	8.66	6.59	7.86	26.01	10.18	19.15	19.91	122.48	115.78	118.54	
MRI335	3.25	5.44	4.43	5.66	9.75	16.80	5.17	1.83	1.42	2.64	2.53	
MRI347	28.29	10.62	11.11	13.80	30.50	14.79	29.15	55.87	25.94	38.95	50.75	33.22
MRI353	6.66	2.90	3.29		8.85	4.22	2.81	4.18	3.18	4.97	3.19	3.23
MRI346	90.13	40.69	28.50	20.56	49.80	54.92	38.21	69.65	37.90	27.57	24.28	29.05
MRI348	17.08	2.79	4.32	4.56	76.31	26.68	16.03	18.54	21.54			
MRI333	6.94	6.56	9.52	7.55	36.93	6.50	13.42	18.36	77.76	25.48	25.00	22.39
MRI317	10.13	3.44	24.51	36.99	37.28	14.15	35.98	47.06	54.70	26.28	45.10	
MRI332	11.73	8.38	3.20	17.65	147.96	25.25	23.12	29.39				
MRI331	13.69	1.66	7.13	3.94	114.99	19.97	47.63	15.51	12.85	11.61	11.67	
MRI360	1.69	2.00	3.16	38.67	53.85	26.97	6.46	8.76	4.24	10.11	10.71	15.11
MRI340	12.70	4.37	10.39	4.69	115.28	102.24	78.82					
MRI323	34.16	5.16	10.16	7.02	30.00	10.10	8.34	11.58	23.05			
MRI358	29.70	4.89	71.38	24.41	88.06	50.42	85.54	70.30	55.83	54.66	58.58	41.23
MRI318	6.12	3.51	5.98	15.34	120.63	17.10	17.74					
MRI339	1.37	1.87	2.31	1.22	7.42	7.12	2.82	2.22	7.55	6.23	3.83	3.91
MRI349	1.78	3.26	4.77	1.03	7.06	6.36	2.41	4.53	4.05	4.48	1.68	0.38
MRI351	16.51	17.25	23.60	33.72		35.29	99.51		59.49	54.82	58.89	54.02
MRI336	2.52	4.64	2.66	2.87	79.57	8.05	6.03	3.82	4.34	6.76	2.46	2.22
MRI338	2.96	3.49	4.23	6.49	9.33	41.22	7.89	41.12	22.43	32.14	32.57	20.36
Comment		Winter		Winter		Winter		Winter		Winter	Post skin test	
Mean	18.05	12.51	16.38	14.65	52.04	25.70	29.17	28.50	33.32	27.97	27.64	17.52
Median	11.73	5.03	7.13	7.55	36.32	16.80	16.03	18.45	21.99	12.66	11.75	11.41
Midrange	45.75	43.66	36.69	24.53	77.51	53.23	56.94	41.31	61.95	59.21	60.11	27.20

Table S4a: Heat-map analysis of results with ST123. Animals marked in yellow in column 1 are those with two or more positive culture results

TP	1	2	3	4	5	6	7	8	9	10	10A
2	0.219										
3	0.823	0.412									
4	0.901	0.319	0.905								
5	0.001	0.000	0.000	0.001							
6	0.118	0.005	0.070	0.105	0.115						
7	0.218	0.011	0.115	0.148	0.073	0.850					
8	0.139	0.006	0.081	0.115	0.115	0.964	0.900				
9	0.132	0.006	0.078	0.115	0.132	0.977	0.850	0.959			
10	0.219	0.015	0.117	0.155	0.115	0.901	0.953	0.925	0.901		
10A	0.418	0.063	0.247	0.319	0.062	0.683	0.850	0.702	0.683	0.823	
11	0.901	0.219	0.702	0.823	0.015	0.305	0.424	0.319	0.310	0.418	0.698

Table S4b: Kruskal Wallace analysis of data with ST123

Table S5: SMP70

	SMP70											
Serum ID	TP1	TP2	TP3	TP4	TP5	TP6	TP7	TP8	TP9	TP10	TP10A	TP11
MRI316	31.6843	36.7016	52.7720		52.8686	54.5542	40.2133	46.4817	115.2057			
MRI324	102.6426	91.7826	93.1566	86.3834	84.7373	92.7088	71.7528	76.6604	80.6549	75.5717	73.3638	
MRI350	16.4918	13.9985	19.4065	24.1341	41.5873	38.1941	30.4991	38.6682				
MRI361	19.1300	43.8787	36.1617	33.3172	92.6562	100.7989	84.9041	49.8486				
MRI329	69.1848	53.0969	69.6545	60.9280	82.2308	94.0696	70.8617	79.6760	76.1424	82.7224	77.5164	
MRI334	62.5516		61.8680	79.7310	91.1262	105.0695	100.1687	71.9181	83.6374	74.9057	72.9569	83.5797
MRI352	37.6704	49.1854	58.2368	54.0862	81.0317	74.7192	93.7941	78.2972	84.4764	73.7382	70.8616	64.3095
MRI344	53.7735	34.1402	45.4196	41.1448	59.7239	72.6823	58.2303	43.8333	55.3530	57.3410	46.8136	44.6951
MRI341	28.4645	24.4197	33.4821	48.4240	76.7683	43.3251	45.4230	62.7077	30.4338	60.7884	48.2420	43.1362
MRI321	75.3674	59.8613	69.9148	63.8374	83.6939	77.5134	79.5392	75.0240	113.4048	112.7318	112.8588	
MRI335	75.9030	38.3738	54.2096	61.9832	66.9208	83.5084	83.7694	46.3501	48.3759	49.1244	43.1088	
MRI347	92.4120	46.2810	54.4271	73.1075	81.1966	69.2070	79.4803	100.8761	80.3054	93.0811	98.6348	80.1134
MRI353	59.3117	71.4273	93.0811		102.9284	78.0401	95.0914	82.2977	86.9303	67.8298	78.8382	61.4840
MRI346	18.5034	19.7276	26.0374	20.7327	35.2426	28.3207	33.8684	80.2754	46.6411	47.5862	42.0055	39.1461
MRI348	42.6265	26.8085	46.7971	41.0063	104.9086	90.7768	85.6131	86.1862	79.5073			
MRI333	103.8416	73.9555	78.9655	68.8370	92.2156	84.3769	82.3151	66.2181	97.7681	81.8846	76.0282	72.7220
MRI317	92.6171	51.4704	54.0351	64.7929	60.3762	50.9893	50.8483	41.6459	75.4856	50.1971	41.6278	
MRI332	30.7904	27.3793	25.9070	28.8190	81.6784	39.5370	37.8586	32.3313				
MRI331	23.0494	17.8441	32.8016	35.4241	96.8747	65.2089	84.9955	48.4717	51.1340	45.1474	46.0988	
MRI360	17.7525	15.3598	34.8197	29.3749	55.5657	44.7164	33.4021	33.4238	30.4648	34.2665	30.7599	35.2594
MRI340	41.1403	30.0821	43.5989	39.3082	108.8891	83.5214	73.8898					
MRI323	41.4847	32.0937	22.8052	46.9552	39.6526	36.4686	39.0151	39.2862	58.8597			
MRI358	27.9606	17.6315	112.1318	59.0979	123.6773	98.0654	126.0113	107.8888	95.0278	93.3790	101.1249	70.8596
MRI318	27.0556	32.6404	33.2216	35.4048	67.5869	38.3369	35.0173					
MRI339	29.7008	25.1444	38.8473	40.3897	70.8915	68.2128	52.8035	42.3717	49.5362	82.2995	50.0428	39.6707
MRI349	34.3095	58.0455	31.3848	40.8666	54.2789	72.7581	31.9288	49.3983	51.0935	45.3411	35.1328	40.1177
MRI351	25.0661	30.6173	42.9828	46.4066		56.8570	67.4453		55.0725	42.3270	37.9792	42.6251
MRI336	36.9733	21.6311	31.9958	25.6883	96.2893	70.3400	54.7803	44.0443	49.3887	70.9434	39.7137	38.8262
MRI338	75.8184	62.9960	61.9626	94.9357	79.9795	115.9541	74.6194	74.6312	22.1504	18.9201	15.1258	6.6025
Comment		Winter		Winter		Winter		Winter		Winter	Post skin test	
Mean	48.04	39.52	50.35	49.82	77.34	69.96	65.45	61.49	67.38	64.77	58.99	50.88
median	37.67	33.39	45.42	46.41	81.11	72.68	70.86	56.28	67.17	67.83	48.24	43.14
Midrange	60.17	52.89	65.77	57.83	79.46	72.14	78.26	70.11	68.68	65.83	63.99	45.09

Table S5a: Heat-map analysis of results with SMP70. Animals marked in yellow in column 1 are those with two or more positive culture results

TP	1	2	3	4	5	6	7	8	9	10	10A
2	0.354										
3	0.741	0.187									
4	0.741	0.187	0.975								
5	0.000	0.000	0.001	0.001							
6	0.003	0.000	0.012	0.015	0.383						
7	0.022	0.001	0.061	0.067	0.156	0.622					
8	0.063	0.004	0.145	0.156	0.077	0.387	0.755				
9	0.012	0.001	0.032	0.037	0.314	0.823	0.757	0.555			
10	0.032	0.002	0.071	0.077	0.199	0.699	0.961	0.741	0.822		
10A	0.187	0.025	0.329	0.341	0.038	0.218	0.505	0.741	0.341	0.505	
11	0.625	0.187	0.823	0.823	0.011	0.071	0.187	0.329	0.127	0.193	0.555

Table S5b: Kruskal Wallis analysis of data with SMP70

Table S6: ZAM 295

	ZAM295											
Serum ID	TP1	TP2	TP3	TP4	TP5	TP6	TP7	TP8	TP9	TP10	TP10A	TP11
MRI316	43.22	56.74	75.70		94.29	77.27	95.77	87.09	88.10			
MRI324	88.19	88.30	90.91	91.00	92.02	85.02	93.69	92.41	89.49	86.23	89.22	
MRI350	31.64	28.59	42.79	29.91	41.26	36.84	41.27	41.23				
MRI361	53.41	82.77	84.63	50.15	71.00	55.48	62.39	61.15				
MRI329	94.69	94.78	78.45	76.51	97.52	82.46	80.83	91.50	94.72	87.97	87.75	
MRI334	58.17		56.19	71.09	82.14	60.28	71.06	57.50	61.89	60.99	74.60	68.50
MRI352	17.22	23.16	25.49	24.29	60.66	38.03	64.38	71.62	49.61	48.87	57.45	59.33
MRI344	77.49	61.95	22.98	31.76	102.06	82.55	76.71	67.78	41.81	71.97	75.22	35.05
MRI341	72.83	15.74	20.15	21.98	75.34	51.67	48.66	31.51	64.66	34.38	38.89	47.73
MRI321	81.68	52.05	37.45	46.61	76.04	41.43	68.77	55.69	102.48	105.00	104.01	
MRI335	37.21	29.09	25.56	51.31	56.03	36.57	58.32	33.98	44.23	41.05	28.14	
MRI347	79.16	32.56	31.98	44.76	66.91	32.20	65.01	78.39	53.59	67.07	73.03	54.77
MRI353	49.25	16.94	18.74		25.06	15.42	13.83	12.25	17.13	26.18	24.98	17.27
MRI346	101.16	73.27	75.93	52.42	76.48	73.35	67.99	94.92	69.17	63.64	61.87	66.81
MRI348	46.18	11.97	19.52	32.06	90.14	47.34	48.63	41.67	33.71			
MRI333	95.02	83.62	88.79	75.48	94.34	60.56	87.00	91.31	104.27	94.91	98.21	95.59
MRI317	94.54	71.54	88.43	99.06	94.19	94.39	94.64	97.35	94.86	94.35	96.16	
MRI332	78.98	59.25	26.32	62.30	100.72	69.13	82.45	78.34				
MRI331	72.97	14.19	61.59	31.30	99.70	81.53	95.33	74.35	76.16	65.70	66.03	
MRI360	23.62	14.61	14.34	73.42	91.53	72.67	32.95	40.16	30.31	34.77	40.68	58.95
MRI340	71.87	25.57	48.47	16.78	94.80	97.38	99.74					
MRI323	89.09	40.62	68.93	49.25	87.43	52.23	50.56	53.53	59.32			
MRI358	79.59	25.00	101.57	59.09	102.64	85.99	104.75	102.99	92.34	94.98	97.11	79.88
MRI318	41.24	41.78	51.11	85.16	101.22	54.99	76.90					
MRI339	7.51	4.46	9.74	8.27	24.71	23.29	13.31	13.16	29.19	12.54	15.16	19.80
MRI349	60.88	58.11	51.53	62.49	87.42	37.74	36.38	53.49	37.36	35.72	29.26	19.42
MRI351	97.54	70.90	68.62	102.23		96.29	101.34		95.56	100.99	102.17	101.95
MRI336	19.77	30.90	13.16	10.99	102.22	71.04	50.89	55.81	50.73	4.80	13.72	10.57
MRI338	6.20	2.03	16.50	37.69	42.50	79.92	51.62	104.88	84.19	91.10	95.72	78.69
Comment		Winter		Winter		Winter		Winter		Winter	Post skin test	
Mean	61.04	43.23	48.81	51.75	79.66	61.83	66.73	64.77	65.20	63.01	65.21	54.29
median	71.87	36.59	48.47	50.15	88.79	60.56	67.99	64.46	63.28	65.70	73.03	58.95
Midrange	53.68	48.41	55.66	55.25	63.67	56.40	59.03	58.57	60.70	54.90	58.86	56.26

Table S6a: Heat-map analysis of results with ZAM295. Animals marked in yellow in column 1 are those with two or more positive culture results

TP	1	2	3	4	5	6	7	8	9	10	10A
2	0.094										
3	0.224	0.737									
4	0.404	0.532	0.908								
5	0.083	0.000	0.001	0.004							
6	0.988	0.094	0.224	0.404	0.083						
7	0.737	0.041	0.094	0.163	0.210	0.737					
8	0.867	0.057	0.149	0.224	0.157	0.867	0.921				
9	0.840	0.057	0.149	0.220	0.185	0.840	0.932	0.961			
10	0.921	0.094	0.220	0.341	0.149	0.921	0.884	0.932	0.922		
10A	0.804	0.057	0.149	0.220	0.220	0.804	0.961	0.932	0.961	0.917	
11	0.735	0.443	0.786	0.912	0.057	0.735	0.394	0.486	0.464	0.609	0.443

Table S6b: Kruskal Wallis analysis of data with ZAM295

Table S7: JJRR121

JRRR121												
Serum ID	TP1	TP2	TP3	TP4	TP5	TP6	TP7	TP8	TP9	TP10	TP10A	TP11
MRI316	3.12	-0.98	0.54		6.20	17.00	-0.97	-0.13	1.10			
MRI324	1.30	1.17	3.95	7.02	14.12	8.56	2.00	0.70	2.87	1.54	0.59	
MRI350	0.32	-1.50	-2.32	-1.43	4.51	2.07	-1.15	0.76				
MRI361	7.99	3.69	0.06	-0.12	6.85	8.69	0.60	6.09				
MRI329	6.17	1.07	0.92	2.50	6.70	8.07	3.02	10.94	10.17	10.22	3.46	
MRI334	1.60		0.82	1.20	3.38	2.14	-0.96	-0.87	9.39	1.53	0.45	0.06
MRI352	1.93	0.17	1.02	5.39	1.27	1.78	1.11	5.21	5.51	2.70	2.08	5.44
MRI344	17.78	15.14	26.59	19.31	35.20	42.78	16.81	21.33	20.32	24.21	15.69	18.10
MRI341	20.82	7.41	14.85	15.85	36.60	18.29	12.67	25.39	3.34	22.35	11.80	9.34
MRI321	10.73	9.67	17.42	9.83	22.57	17.96	6.99	11.23	13.66	16.29	9.27	
MRI335	17.02	11.11	15.14	10.76	30.72	43.62	18.71	17.19	15.10	42.44	19.37	
MRI347	15.04	11.21	19.94	13.92	14.68	19.70	9.07	17.66	22.55	15.23	8.66	13.14
MRI353	9.45	6.04	14.18		31.27	22.03	10.32	12.34	11.56	23.04	13.71	11.81
MRI346	13.57	8.14	20.98	9.36	31.03	18.78	10.25	16.56	14.18	24.80	14.49	8.66
MRI348	12.37	5.86	8.45	5.75	23.02	17.98	12.66	12.38	14.23			
MRI333	15.44	3.24	5.87	2.50	20.68	28.07	8.05	5.95	30.06	18.93	12.78	7.62
MRI317	3.53	1.91	2.83	3.76	13.04	8.09	8.69	7.11	17.12	9.36	7.20	
MRI332	6.24	19.71	12.12	5.99	32.70	8.33	4.21	2.34				
MRI331	9.01	2.58	5.14	4.26	14.40	8.22	4.34	3.36	6.56	4.34	4.10	
MRI360	3.40	1.42	4.30	3.20	1.27	-0.09	3.21	2.05	3.88	8.05	8.67	4.06
MRI340	3.14	5.05	19.87	17.41	38.06	51.57	31.68					
MRI323	4.85	2.76	3.41	3.34	11.15	16.22	5.35	4.62	8.22			
MRI358	3.87	2.57	4.26	3.88	16.59	15.27	5.08	8.95	8.23	11.19	9.19	9.13
MRI318	4.75	-0.47	4.77	2.88	16.43	2.78	-2.59					
MRI339	-3.07	-0.86	0.49	-0.28	2.83	6.70	-0.79	-0.46	0.58	2.35	1.82	3.26
MRI349	2.70	5.24	1.51	-0.23	3.06	11.47	17.65	7.10	7.55	11.28	10.38	8.36
MRI351	3.46	3.99	15.05	6.10		17.57	11.65		4.90	4.05	2.73	2.02
MRI336	6.81	4.48	7.92	7.76	9.84	9.86	3.53	2.86	5.92	13.11	4.57	5.10
MRI338	3.33	4.40	0.92	0.70	7.80	55.61	1.39	1.80	3.33	0.93	2.98	1.67
Comment		Winter		Winter		Winter		Winter		Winter	Post skin test	
Mean	4.85	3.84	4.77	4.26	14.26	15.27	5.08	6.02	8.23	11.19	8.66	7.62
Median	4.85	3.84	4.77	4.26	14.26	15.27	5.08	6.02	8.23	11.19	8.66	7.62
Midrange	8.87	9.10	12.13	8.94	19.67	27.76	14.55	12.26	15.32	21.68	9.91	9.08

Table S7a: Heat-map analysis of results with JRRR121. Animals marked in yellow in column 1 are those with two or more positive culture results

TP	1	2	3	4	5	6	7	8	9	10	10A
2	0.326										
3	0.985	0.326									
4	0.635	0.672	0.637								
5	0.023	0.001	0.023	0.006							
6	0.023	0.001	0.023	0.006	0.972						
7	0.819	0.503	0.821	0.828	0.011	0.011					
8	0.972	0.326	0.972	0.621	0.030	0.030	0.819				
9	0.355	0.035	0.355	0.142	0.326	0.326	0.227	0.396			
10	0.202	0.017	0.202	0.059	0.613	0.621	0.096	0.227	0.774		
10A	0.819	0.227	0.819	0.475	0.096	0.096	0.621	0.828	0.621	0.355	
11	0.925	0.334	0.925	0.621	0.096	0.096	0.774	0.938	0.604	0.355	0.938

Table S7b: Kruskal Wallis analysis of data with JRRR121

Table S8: ID Screen®

	ID Screen®											
Serum ID	TP1	TP2	TP3	TP4	TP5	TP6	TP7	TP8	TP9	TP10	TP10A	TP11
MRI316	8	19	36	31	20	18	10	9	14			
MRI324	3	6	5	11	12	8	7	11	11	-1	1	2
MRI350	2	9	3	3	5	4	10	179				
MRI361	7	27	10	29	54	69	257	179				
MRI329	7	8	8	7	11	12	5	10	14	0	17	
MRI334	11	8	6	7	7	9	6	5	11	-3	21	-1
MRI352	7	20	18	16	6	7	3	9	17	-4	-1	
MRI344	6	16	40	22	16	8	3	3	3	-5	0	-2
MRI341	12	7	8	8	14	14	5	4	6	6	-4	-1
MRI321	6	6	8	32	7	9	2	2	12	-4	29	-2
MRI335	3	9	12	10	4	9	3	2	5	-4	-1	-2
MRI347	8	6	23	11	10	8	4	16	11	-1	8	1
MRI353	4	6	7		5	3	3	4	8	-2	3	4
MRI346	6	10	13	12	6	8	5	4	15	-4	8	0
MRI348	5	5	5	6	25	10	5	35	32			
MRI333	26	20	19	13	23	18	9	19	62	11	106	13
MRI317	10	12	7	9	14	11	7	8	9	-1	22	0
MRI332	6	31	16	12	73	17	4	6				0
MRI331	5	8	5	11	6	6	6	7	-3	-1	-1	
MRI360	1	5	6	3	26	22	37	18		-1	3	3
MRI340	5	10	12	23	66	28	7					
MRI323	6	8	4	16	7	6	5	4	8			
MRI358	3	9	5	8	7	10	4	4	6	-4	1	-2
MRI318	2	12	7	11	292	26	3					
MRI339	25	13	12	14	8	7	3	5	22	-2	1	0
MRI349	10	16	11	8	16	8	3	7	12	-2	25	2
MRI351	5	4	7	11	119	65	60	42	54	21	41	14
MRI336	6	2	5	4	34	31	2	3	5	-5	-2	2
MRI338	8	6	8	13	13	14	3	8	6	-3	-2	1
Comment		Winter		Winter		Winter		Winter		Winter	Post skin test	
Mean	7.34	10.97	11.24	12.89	31.24	16.03	16.59	22.33	14.78	-0.43	13.10	1.78
Median	6	9	8	11	13	10	5	7	11	-2	3	0.5
Midrange	13.5	16.5	21.5	17.5	148	36	129.5	90.5	29.5	8	51	6

Table S8a: Reported data for IDScreen® [40].

TP	1	2	3	4	5	6	7	8	9	10	10A
2	0.701										
3	0.988	0.717									
4	0.463	0.735	0.467								
5	0.124	0.053	0.119	0.021							
6	0.319	0.558	0.331	0.803	0.010						
7	0.266	0.111	0.263	0.053	0.746	0.022					
8	0.111	0.266	0.115	0.463	0.002	0.622	0.006				
9	0.090	0.029	0.087	0.014	0.790	0.006	0.558	0.002			
10	0.392	0.191	0.384	0.104	0.691	0.053	0.922	0.016	0.505		
10A	0.443	0.691	0.455	0.922	0.022	0.922	0.053	0.558	0.016	0.104	
11	0.988	0.733	0.988	0.505	0.171	0.384	0.319	0.153	0.111	0.440	0.467

Table S8b: Kruskal Wallis analysis of data with IDScreen®.

Table S9: PPDA IGRA

	PPDA											
Serum ID	TP1	TP2	TP3	TP4	TP5	TP6	TP7	TP8	TP9	TP10	TP10A	TP11
MRI316	4.8	5.3	2.6	12.2	3.3	59.2	1.5	9.7	2.1			
MRI324	1.1	3.3	0.9	1.8	0	0.2	0.8	0	0.2	0.3	5.2	0.8
MRI350	1	6.2	3	4.2	6	7	1.4	11.4				
MRI361	0.6	4.3	1.8	4.8	2	4.5	1.2	5.2				
MRI329	4.5	6.4	0.7	5.2	1.5	5.5	2.7	13.2	0.5	4.3	24.9	2.5
MRI334	1.9	4.4	3.6	10.7	0.9	4.5	1.2	4.1	0.7	0.5	6.2	0.1
MRI352	6.5	5.7	8.7	11.5	4.8	67.4	4.9	26.2	1.2	17.6	26.7	6.5
MRI344	1.1	1.6	1	2.4	0.1	1.6	0.5	3.3	1	1.6	3.6	0.9
MRI341	1.9	3.9	2	4.8	0	4.9	0.5	2.4	0.7	0.7	26.4	2.6
MRI321	0.7	0.3	1.8	3.3	1.1	2.2	2.6	2.8	0.6	0.7	4.1	1.7
MRI335	3.7	2.5	1	4.6	0.7	3	0.5	1.3	0.7	1.6	21.4	5.3
MRI347	3.1	6.7	2	1.8	1.7	1.3	4.6	3.8	1.3	4.7	12	3.9
MRI353	2.8	2.5	3		1.6	10.2	2.2	3.2	1.7	3	7.4	4.3
MRI346	0.7	2.3	2.9	1.6	0.8	2	0	2.6	0.6	0.1	5.4	1.3
MRI348	0.6	1.5	2.3	0.9	0.2	19.2	0	3.8	1.2			
MRI333	2.8	2.9	2.2	1.7	1.3	10.1	4.6	3.9	0.6	0.9	4.8	4.7
MRI317	2.5	0.8	1.4	6.3	0.5	7.7	0	5	0.9	1.6	2.3	1.8
MRI332	1.1	0.5	0.3	0.2	0.2	1.6	0.9	0				
MRI331	2.5	3.7	5	16.8	0.9	6.4	0.9	8.8	0.3	29.8	6.3	
MRI360	2.1	12.2	3.7	10.7	1.4	36	0.3	10.4	0	3	9.3	3.9
MRI340	0.9	0.9	0.4	2.4	0.7	4.3	3.3					
MRI323	3.3	2.1	2	5.6	1.3	5.2	0.9	3.8	4.7			
MRI358	7.2	12.2	4.4	7.5	4.2	16.7	3	15.4	2.2	3.4	99.6	24.7
MRI318	10.5	12.7	7.9	49.7	1.1	28.6	1					
MRI339	2.9	11.1	2.5	3.5	1.3	1.4	3	5.2	0.9	0.3	9.6	1.2
MRI349	10	6.4	9.3	27.7	2.6	7	4.8	8.2	0.2	11.2	18.1	8.4
MRI351	0.7	1.5	3.4	4.2	2.5	10.2	3.4	8.4	1.3	2.1	99.4	7.46
MRI336	4.3	1.5	1.8	5.5	0.9	4.7	0.1	6.3	0.2	2.2	1.2	0.3
MRI338	3.2	1.8	1	2.8	0.3	0.9	4.39	1.4	0.2	1.2	2.1	0.5
Comment		Winter		Winter		Winter		Winter		Winter	Post skin test	
Mean	3.07	4.39	2.85	7.66	1.51	11.50	1.90	6.29	1.00	4.32	18.86	4.14
median	2.5	3.3	2.2	4.7	1.1	5.2	1.2	4.1	0.7	1.6	7.4	2.55
Midrange	5.55	6.5	4.8	24.95	3	33.8	2.45	13.1	2.35	14.95	50.4	12.4

Table S9a: Heat-map analysis of results with PPDA IGRA. Animals marked in yellow in column 1 are those with two or more positive culture results

TP	1	2	3	4	5	6	7	8	9	10	10A
2	0.27										
3	0.94	0.23									
4	0.03	0.29	0.02								
5	0.04	0.00	0.04	0.00							
6	0.01	0.16	0.01	0.76	0.00						
7	0.15	0.01	0.17	0.00	0.54	0.00					
8	0.03	0.32	0.03	0.95	0.00	0.73	0.00				
9	0.00	0.00	0.00	0.00	0.38	0.00	0.14	0.00			
10	0.68	0.15	0.73	0.01	0.15	0.01	0.39	0.02	0.03		
10A	0.00	0.02	0.00	0.16	0.00	0.27	0.00	0.15	0.00	0.00	
11	0.81	0.45	0.76	0.09	0.03	0.04	0.14	0.10	0.00	0.54	0.00

Table S9b: Kruskal Wallis analysis of data with PPDA IGRA

Table S10: MAP 3651c_IGRA

	3651c											
Serum ID	TP1	TP2	TP3	TP4	TP5	TP6	TP7	TP8	TP9	TP10	TP10A	TP11
MRI316	1.7	1.7	0.7	4.7	0.92	14.2	0	5.8	0.8			
MRI324	0.7	1.1	0.1	0.5	0.1	0	0.5	0	0	0.3	0.3	0.3
MRI350	0.3	1.7	0.8	1	0.8	1.3	0	4.9				
MRI361	0	0	0.3	1.2	0.5	0.2	0.4	3				
MRI329	0.3	1.2	0.4	0	0.2	0	0	3	0.2	0.2	0.9	0.7
MRI334	0.5	1.3	1	0.2	0.6	1.4	0.3	1.1	0	0.2	1.2	0.1
MRI352	1.1	0.1	1.7	0.5	0	1.1	1.5	10.8	0	1	0.1	0.24
MRI344	0.1	0.4	0	0.2	0	0	0	0	0	0.1	0.2	0.2
MRI341	0.8	2.32	0.6	0.7	0	1.1	0	0.57	0.2	0.2	0.7	0.3
MRI321	0	0	0	1.1	0	0.3	0.4	0	0.3	0	0.1	0.1
MRI335	0.8	1.1	1	1.4	0	0.5	0	0	0.2	0.6	2	1.7
MRI347	0.1	1.2	0.5	0.7	0.3	0.3	1	2	0.3	0.3	1.5	0.9
MRI353	0.4	1.09	1.6		0.5	2.9	0	1.1	0.3	1.1	0.2	1.1
MRI346	0	0	0.3	0.7	0	0	0	0.6	0.1	0	0.3	0.1
MRI348	0	0	0.4	0	0	1.7	0.2	1.7	1.1			
MRI333	0.9	0.2	0	0.3	0	2.1	0.7	0	0	0	0	1.1
MRI317	0.3	0	0	1.5	0.2	0.6	0	1.3	0.2	0.1	0.4	0.1
MRI332	0.8	0	0.1	0	0	0.4	0.2	0				
MRI331	0.6	1.6	1.8	3.3	0.4	6.2	0.3	4.1	0.1	2.4	3.6	
MRI360	0.2	2.9	1.2	5.3	1.134	6.9	0.1	3.5	0	0.1	1.9	0
MRI340	0.2	0.3	0.2	0	0.7	1.1	1.2					
MRI323	1.5	0	0.8	1.4	0.3	0.8	0	0.2	0.7			
MRI358	0	0.8	0.1	0.6	0.6	1.1	0.4	4.5	0.5	0	6.4	3.4
MRI318	0.5	0.9	0.8	1.8	0	1.2	0					
MRI339	0.3	2.5	0.1	0.2	0	0.6	1.2	2.3	0	0.1	0.4	0
MRI349	1.8	0.6	1.2	0	0.4	0	2.3	3.9	0	0.6	1	2.1
MRI351	0.4	0.2	0.3	1.2	0.6	2.4	1.4	4.2	0.9	0.3	5.9	1.6
MRI336	1.6	0.6	0.4	9.5	0	1	0.2	4.1	0	0.5	0.3	0.2
MRI338	0.9	0.4	0.3	0.1	0.1	0	0	0	0	0.2	0.2	0.2
Comment		Winter		Winter		Winter		Winter		Winter	Post skin test	
Mean	0.58	0.83	0.58	1.36	0.29	1.70	0.42	2.32	0.25	0.40	1.31	0.72
Median	0.4	0.6	0.4	0.7	0.2	1	0.2	1.7	0.15	0.2	0.4	0.27
Midrange	0.9	1.45	0.9	4.75	0.567	7.1	1.15	5.4	0.55	1.2	3.2	1.7

Table S10a: Heat-map analysis of results with MAP 3651c IGRA. Animals marked in yellow in column 1 are those with two or more positive culture results

TP	1	2	3	4	5	6	7	8	9	10	10A
2	0.701										
3	0.988	0.717									
4	0.463	0.735	0.467								
5	0.124	0.053	0.119	0.021							
6	0.319	0.558	0.331	0.803	0.010						
7	0.266	0.111	0.263	0.053	0.746	0.022					
8	0.111	0.266	0.115	0.463	0.002	0.622	0.006				
9	0.090	0.029	0.087	0.014	0.790	0.006	0.558	0.002			
10	0.392	0.191	0.384	0.104	0.691	0.053	0.922	0.016	0.505		
10A	0.443	0.691	0.455	0.922	0.022	0.922	0.053	0.558	0.016	0.104	
11	0.988	0.733	0.988	0.505	0.171	0.384	0.319	0.153	0.111	0.440	0.467

Table S10b: Kruskal Wallis analysis of data with MAP 3651c IGRA

Figure S2: Assessment of significant differences between Summer and Winter timepoint data.

In order to determine whether there was a systematic difference between the winter and summer responses, the total sets of results were compared for each antigen.

A p-value of less than 0.05 indicates significant difference.

Figure S2.1: Analysis of Variance (One-Way) for PPDA IGRA data

Descriptive Statistics

Groups	*Sample size*	*Sum*	*Variance*	*Std Dev*	*Mean*	*95% Confidence Interval**	
Summer	144	155.3450	1.0334	1.0166	1.0788	0.9113	1.2462
Winter	181	722.5800	45.6893	6.7594	3.9922	3.0008	4.9835
Total	325		27.9400	5.2858	2.7013		

Confidence intervals are calculated using individual standard deviations.

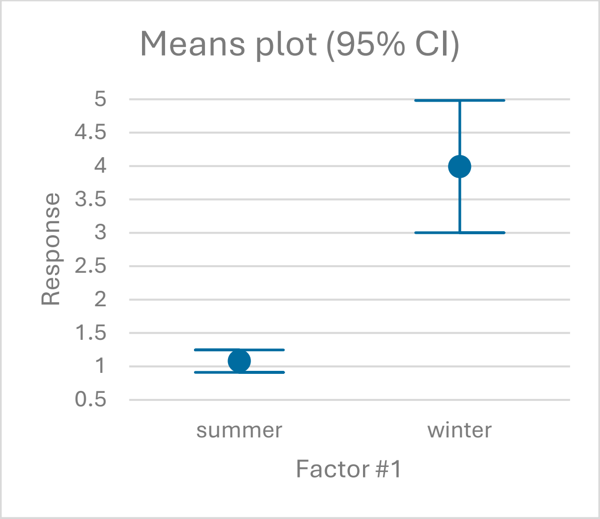

ANOVA

Source of Variation	*d.f.*	*SS*	*MS*	*F*	*p-value*	*F crit*	*Omega Sqr.*
Between Groups	1	680.6894	680.6894	26.2621	5.1409E-7	3.8704	0.0721
Within Groups	323	8,371.8560	25.9191				
Total	324	9,052.5454					

Figure S2.2: Analysis of Variance (One-Way) for MAP 3651c IGRA data

Descriptive Statistics

Groups	*Sample size*	*Sum*	*Variance*	*Std Dev*	*Mean*	*95% Confidence Interval**	
Summer	144	31.0270	0.0598	0.2445	0.2155	0.1752	0.2557
Winter	181	115.7600	0.9565	0.9780	0.6396	0.4961	0.7830
Total	325		0.6023	0.7761	0.4517		

Confidence intervals are calculated using individual standard deviations.

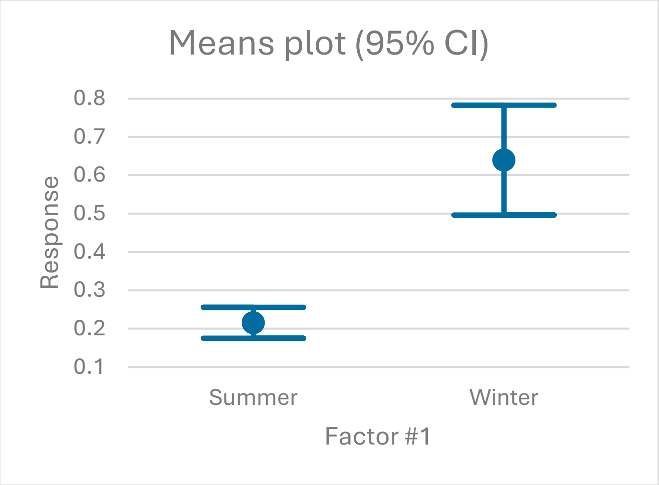

ANOVA

Source of Variation	*d.f.*	*SS*	*MS*	*F*	*p-value*	*F crit*	*Omega Sqr.*
Between Groups	1	14.4238	14.4238	25.7791	6.480E-7	3.8704	0.0708
Within Groups	323	180.7234	0.5595				
Total	324	195.1472					

Figure S2.3: Analysis of Variance (One-Way) for ID Screen® data

Descriptive Statistics

Groups	*Sample size*	*Sum*	*Variance*	*Std Dev*	*Mean*	*95% Confidence Interval**	
Summer	144	2,318.0000	1,196.3961	34.5890	16.0972	10.3996	21.7949
Winter	179	2,092.0000	491.2387	22.1639	11.6872	8.4180	14.9563
Total	323		807.6931	28.4199	13.6533		

Confidence intervals are calculated using individual standard deviations.

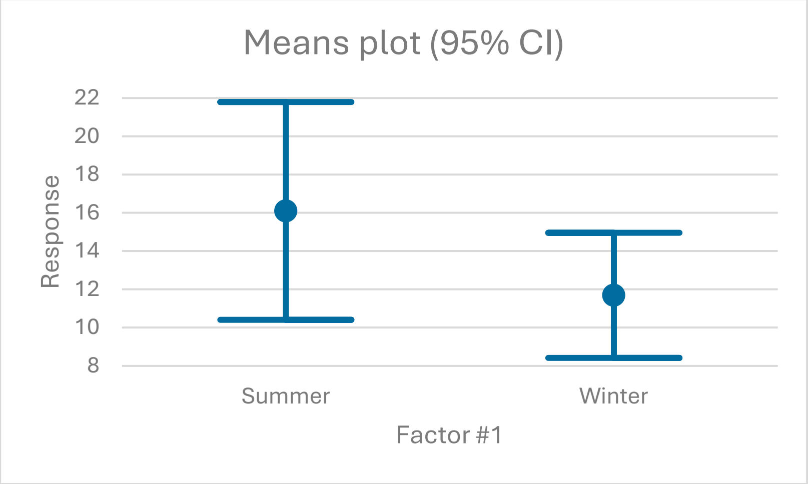

ANOVA

Source of Variation	*d.f.*	*SS*	*MS*	*F*	*p-value*	*F crit*	*Omega Sqr.*
Between Groups	1	1,552.0448	1,552.0448	1.9271	0.1660	3.8706	0.0029
Within Groups	321	258,525.1193	805.3742				
Total	322	260,077.1641					

Figure S2.4: Analysis of Variance (One-Way) for MOD171 data

Descriptive Statistics

Groups	*Sample size*	*Sum*	*Variance*	*Std Dev*	*Mean*	*95% Confidence Interval**	
Summer	144	4,769.6819	822.6639	28.6821	33.1228	28.3981	37.8474
Winter	174	5,110.1451	670.6721	25.8973	29.3686	25.4936	33.2437
Total	318		740.6236	27.2144	31.0686		

Confidence intervals are calculated using individual standard deviations.

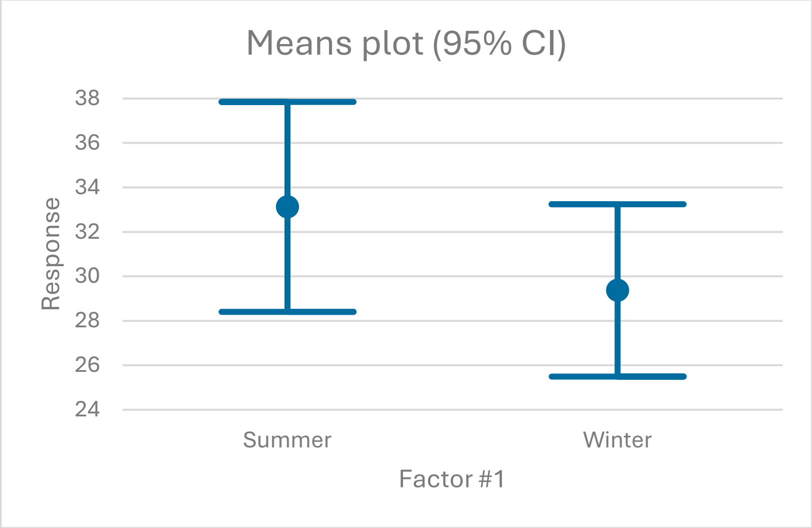

ANOVA

Source of Variation	*d.f.*	*SS*	*MS*	*F*	*p-value*	*F crit*	*Omega Sqr.*
Between Groups	1	1,110.4675	1,110.4675	1.5017	0.2213	3.8711	0.0016
Within Groups	316	233,667.2064	739.4532				
Total	317	234,777.6739					

Figure S2.5: Analysis of Variance (One-Way) for RT237 data

Descriptive Statistics

Groups	*Sample size*	*Sum*	*Variance*	*Std Dev*	*Mean*	*95% Confidence Interval**	
Summer	143	7,285.4419	893.9996	29.8998	50.9471	46.0044	55.8899
Winter	172	8,490.5596	1,021.9932	31.9686	49.3637	44.5521	54.1754
Total	315		961.4795	31.0077	50.0825		

Confidence intervals are calculated using individual standard deviations.

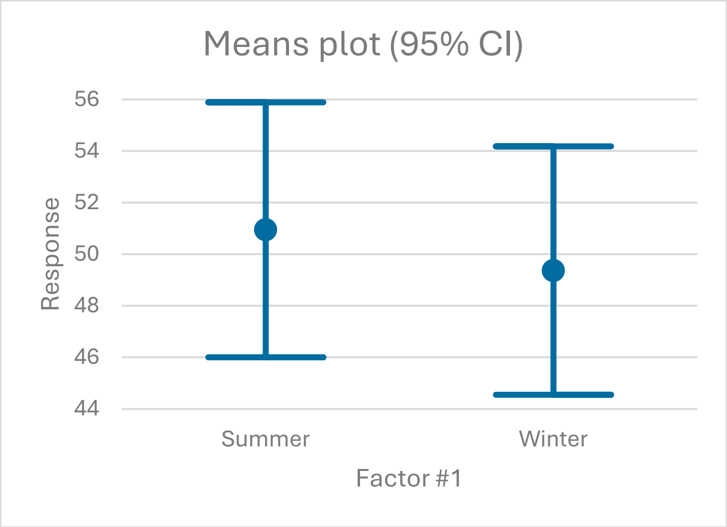

ANOVA

Source of Variation	*d.f.*	*SS*	*MS*	*F*	*p-value*	*F crit*	*Omega Sqr.*
Between Groups	1	195.7719	195.7719	0.2031	0.6525	3.8713	-0.0025
Within Groups	313	301,708.7765	963.9258				
Total	314	301,904.5484					

Figure S2.6: Analysis of Variance (One-Way) for ST123 data

Descriptive Statistics

Groups	*Sample size*	*Sum*	*Variance*	*Std Dev*	*Mean*	*95% Confidence Interval**	
Summer	143	4,261.0338	1,015.1466	31.8614	29.7974	24.5305	35.0644
Winter	172	3,893.0201	552.1353	23.4976	22.6338	19.0972	26.1705
Total	315		772.5253	27.7943	25.8859		

Confidence intervals are calculated using individual standard deviations.

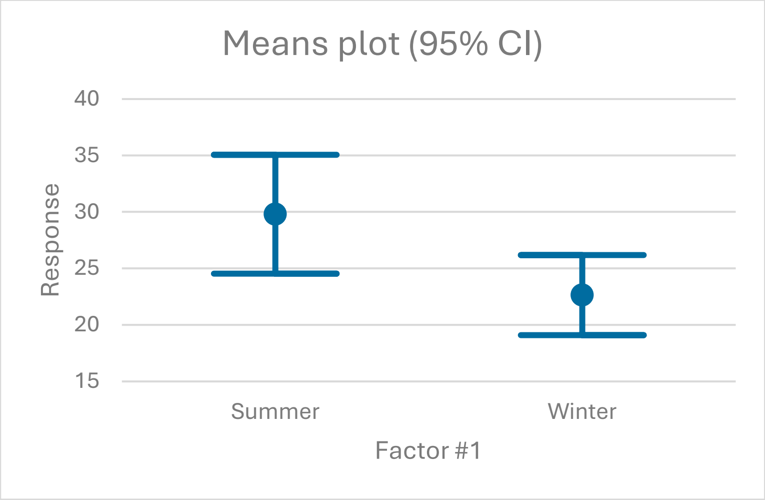

ANOVA

Source of Variation	*d.f.*	*SS*	*MS*	*F*	*p-value*	*F crit*	*Omega Sqr.*
Between Groups	1	4,006.9766	4,006.9766	5.2572	0.0225	3.8713	0.0133
Within Groups	313	238,565.9659	762.1916				
Total	314	242,572.9425					

Figure S2.7: Analysis of Variance (One-Way) for JRRR121 data

Descriptive Statistics

Groups	*Sample size*	*Sum*	*Variance*	*Std Dev*	*Mean*	*95% Confidence Interval**	
Summer	143	1,348.6512	79.9208	8.9398	9.4311	7.9533	10.9090
Winter	173	1,519.6848	89.4067	9.4555	8.7843	7.3653	10.2033
Total	316		84.9507	9.2169	9.0770		

Confidence intervals are calculated using individual standard deviations.

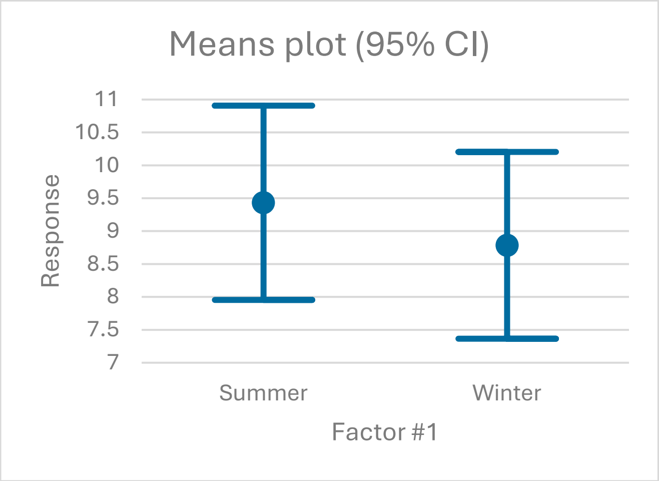

ANOVA

Source of Variation	*d.f.*	*SS*	*MS*	*F*	*p-value*	*F crit*	*Omega Sqr.*
Between Groups	1	32.7540	32.7540	0.3848	0.5355	3.8712	-0.0020
Within Groups	314	26,726.7163	85.1169				
Total	315	26,759.4704					

Figure S2.8: Analysis of Variance (One-Way) for SMP70 data

Descriptive Statistics

Groups	*Sample size*	*Sum*	*Variance*	*Std Dev*	*Mean*	*95% Confidence Interval**	
Summer	143	8,535.3892	699.2221	26.4428	59.6880	55.3168	64.0593
Winter	173	9,532.7218	568.3639	23.8404	55.1024	51.5247	58.6801
Total	316		630.7757	25.1152	57.1776		

Confidence intervals are calculated using individual standard deviations.

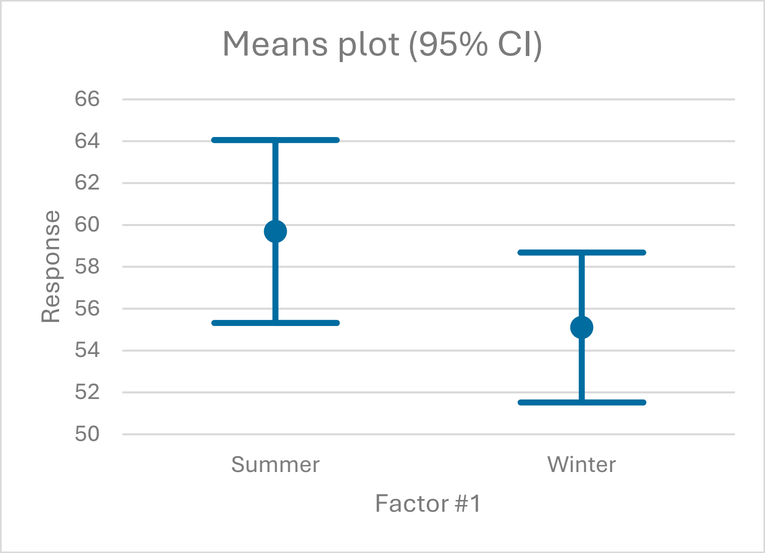

ANOVA

Source of Variation	*d.f.*	*SS*	*MS*	*F*	*p-value*	*F crit*	*Omega Sqr.*
Between Groups	1	1,646.2169	1,646.2169	2.6233	0.1063	3.8712	0.0051
Within Groups	314	197,048.1280	627.5418				
Total	315	198,694.3448					

Figure S2.9: Analysis of Variance (One-Way) for ZAM295 data

Descriptive Statistics

Groups	*Sample size*	*Sum*	*Variance*	*Std Dev*	*Mean*	*95% Confidence Interval**	
Summer	143	9,263.8811	767.0835	27.6963	64.7824	60.2039	69.3608
Winter	173	10,036.8656	775.4242	27.8464	58.0166	53.8377	62.1955
Total	316		780.5795	27.9389	61.0783		

Confidence intervals are calculated using individual standard deviations.

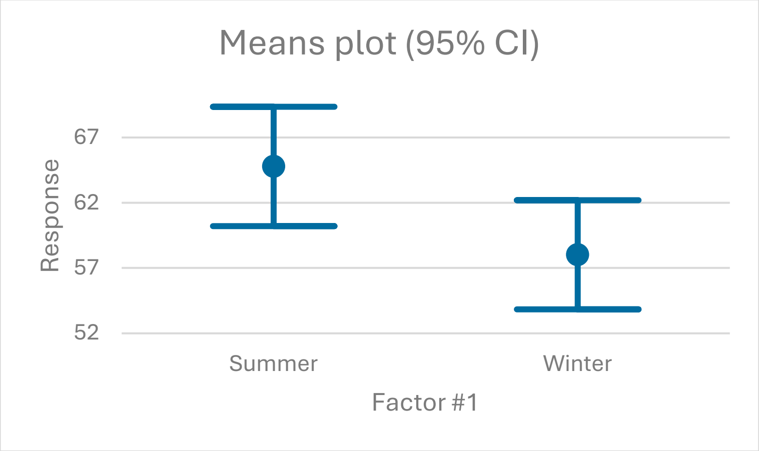

ANOVA

Source of Variation	*d.f.*	*SS*	*MS*	*F*	*p-value*	*F crit*	*Omega Sqr.*
Between Groups	1	3,583.7370	3,583.7370	4.6442	0.0319	3.8712	0.0114
Within Groups	314	242,298.8211	771.6523				
Total	315	245,882.5581					

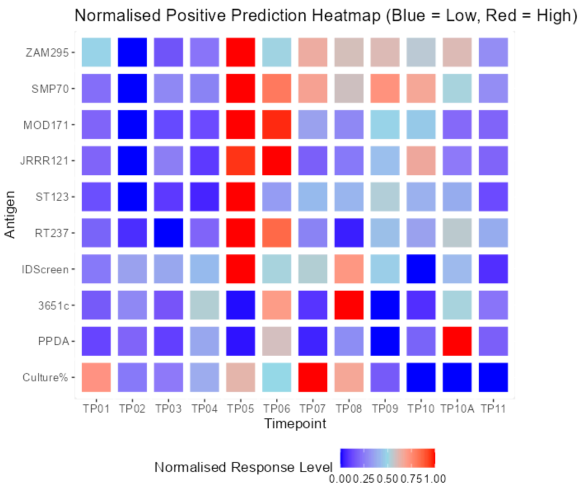

Figure S3: Colour representation of changes in mean response in each assay over time. Each assay is separately normalized. For the culture results, each positive at a particular time is counted as 1, and each negative is counted as 0.
